# Genomic Prediction of Agronomic Traits in Common Bean (*Phaseolus vulgaris* L.) Under Environmental Stress

**DOI:** 10.3389/fpls.2020.01001

**Published:** 2020-07-07

**Authors:** Beat Keller, Daniel Ariza-Suarez, Juan de la Hoz, Johan Steven Aparicio, Ana Elisabeth Portilla-Benavides, Hector Fabio Buendia, Victor Manuel Mayor, Bruno Studer, Bodo Raatz

**Affiliations:** ^1^Bean Program, Agrobiodiversity Area, International Center for Tropical Agriculture (CIAT), Cali, Colombia; ^2^Molecular Plant Breeding, Institute of Agricultural Sciences, ETH Zurich, Zurich, Switzerland

**Keywords:** genomic selection, genotype × environment interactions, common bean (*Phaseolus vulgaris* L.), genome-wide association studies (GWAS), plant breeding, drought, low phosphorus stress

## Abstract

In plant and animal breeding, genomic prediction models are established to select new lines based on genomic data, without the need for laborious phenotyping. Prediction models can be trained on recent or historic phenotypic data and increasingly available genotypic data. This enables the adoption of genomic selection also in under-used legume crops such as common bean. Beans are an important staple food in the tropics and mainly grown by smallholders under limiting environmental conditions such as drought or low soil fertility. Therefore, genotype-by-environment interactions (G × E) are an important consideration when developing new bean varieties. However, G × E are often not considered in genomic prediction models nor are these models implemented in current bean breeding programs. Here we show the prediction abilities of four agronomic traits in common bean under various environmental stresses based on twelve field trials. The dataset includes 481 elite breeding lines characterized by 5,820 SNP markers. Prediction abilities over all twelve trials ranged between 0.6 and 0.8 for yield and days to maturity, respectively, predicting new lines into new seasons. In all four evaluated traits, the prediction abilities reached about 50–80% of the maximum accuracies given by phenotypic correlations and heritability. Predictions under drought and low phosphorus stress were up to 10 and 20% improved when G × E were included in the model, respectively. Our results demonstrate the potential of genomic selection to increase the genetic gain in common bean breeding. Prediction abilities improved when more phenotypic data was available and G × E could be accounted for. Furthermore, the developed models allowed us to predict genotypic performance under different environmental stresses. This will be a key factor in the development of common bean varieties adapted to future challenging conditions.

## Introduction

Common bean (*Phaseolus vulgaris* L.) is an important protein source for human nutrition contributing to food security in the tropics (Broughton et al., 2003). Its affordable and nutrient-rich grains provide protein and micronutrients such as iron and zinc for millions of people in Latin America and eastern/southern Africa, where consumption can reach up to 66 kg per capita annually (Broughton et al., 2003; Blair, 2013). Bean breeding aims to sustainably increase grain production under challenging environmental conditions. An important breeding target is the resistance to biotic and abiotic stresses, such as diseases, drought and low phosphorus (P) (Mukankusi et al., 2018; Assefa et al., 2019). Drought incidences cause up to 70% yield loss (Asfaw et al., 2012; Diaz et al., 2018). Low P and drought constrain bean production in up to 50 and 70% of the area under cultivation, respectively (Wortmann et al., 1998; Beebe et al., 2009). Improving common beans for drought and low soil fertility conditions is therefore of special importance for current breeding programs (Buruchara et al., 2011; Beebe et al., 2013; Mukankusi et al., 2018).

Many traits contribute to drought tolerance and a quantitative breeding approach is required for successful selection (Beebe et al., 2013). For example, early maturing bean lines could avoid drought stress but this strategy potentially affects yield negatively (Kamfwa et al., 2015; Polania et al., 2017). Another strategy is to select for a bigger and deeper root system to increase drought tolerance (Polania et al., 2017). However, increased root biomass under low P conditions decreased shoot biomass (Nielsen et al., 2001). Still, high yielding lines under drought usually perform well under well-watered or low P stress conditions (Beebe et al., 2008; Beebe et al., 2009; Beebe et al., 2013). This indicates that the underlying traits, such as the efficiency of photosynthate translocation to the seeds, may be favorable under most stress conditions (Rao et al., 2017).

Several QTL for seed yield and associated traits have been identified in common bean lines under drought and rain-fed conditions (Mukeshimana et al., 2014; Trapp et al., 2015; Briñez et al., 2017; Hoyos-Villegas et al., 2017; Diaz et al., 2018). However, QTL in different studies differ in their position, and their effects often vary between different years and locations. With a few exceptions: A yield QTL at the lower end of chromosome (Chr) 4 was repetitively found in six out of 15 field trials across different locations under rain-fed conditions in a bi-parental population (Diaz et al., 2018). Two QTL for seed yield on Chr 3 and Chr 9 were found under irrigated conditions (Kamfwa et al., 2015) and under drought conditions (Mukeshimana et al., 2014). Recently, several stable QTL were reported for seed shape and maturity traits but none for seed yield in an extensive analysis of over 600 lines in four environments (Wu et al., 2020). QTL are usually mapped in contrasting individuals for a particular trait. However, mapping and introducing those favorable alleles into breeding material is costly and time consuming. Most successful examples of QTL mapping or genome-wide association studies (GWAS) leading to marker development are for disease resistance (Mukankusi et al., 2018; Gil et al., 2019; Nay et al., 2019). For more complex agronomic traits, genetically determined by many genes, marker-based introgression of a few QTL was not successful.

Genomic selection was developed to predict quantitative traits that are expensive to phenotype and has been successfully applied in animal and plant breeding (Crossa et al., 2017; Georges et al., 2019). The ability to retrieve genomic-estimated breeding values (GEBVs) of new lines reduces phenotyping costs and allows increasing selection intensity in breeding populations. Further benefits include the enrichment of positive alleles in early generations and shortening of breeding cycles by crossing in earlier generations based on the GEBVs (Lehermeier et al., 2017b). In genomic selection, the phenotype is predicted from all genetic markers together (Meuwissen et al., 2001). The problem that number of observations << predictors can be addressed by penalizing the model coefficients as in Ridge regression (Endelman, 2011). The prediction ability (PA) of such a model is dependent on the heritability of the trait, polygenicity of the trait, the ability of markers to tag all informative haplotypes (depending on population structure, linkage disequilibrium and marker density) and the ability to identify the genetic effect of the haplotypes in the population (depending on population size, population structure, and minor allele frequencies) (Kwong et al., 2017; Norman et al., 2018). Several studies have successfully improved PA by adding QTLs as fixed effects to the model. Some examples include field trials in winter wheat (Sarinelli et al., 2019), in a maize panel (Bian and Holland, 2017) and in rice (Spindel et al., 2016). In common bean, a small prediction study including 80 cultivars and 377 molecular markers was able to capture about 25% of the heritable variance in grain yield (Barili et al., 2018). However, the efficacy and usefulness that genomic selection offers for common bean breeding still needs to be assessed in larger and more complete studies that deal with realistic scenarios of the breeding schemes.

Genomic selection in plants must consider strong genotype by environment interactions (G × E), as their effects are usually stronger than in animal production. Season to season prediction can be further improved when models are augmented with environmental covariates (Technow et al., 2015; Oakey et al., 2016). The detection and modelling of G × E was reported to increase the prediction abilities in crop breeding lines up to 20% (Heslot et al., 2014; Acosta-Pech et al., 2017). Such genomic predictions (GPs) under different environments, however, require large training data sets of the targeted populations and environments.

This paper presents the results of applying GP methods to common bean in the context of a breeding program. A panel of elite Andean breeding lines was assessed for different agronomic traits in two locations under drought, irrigated and low P conditions in order to optimize model parameters, characterize G × E effects and simulate breeding applications.

## Material and Methods

### Plant Material

The Andean elite bean breeding (Vivero Equipo Frijol, VEF) panel from the International Center of Tropical Agriculture (CIAT) was evaluated in twelve field trials across two locations in Colombia. The whole set of trials contained 481 different lines. From these, 26 lines are also in the Andean Diversity Panel (ADP) (Cichy et al., 2015). Each trial contained between 156 and 345 lines. These lines were tested in one to twelve trials according to the year of introduction to the panel (**Supplementary Figure 1**). The complete list of lines in the VEF panel is available online (see data availability statement Keller et al., 2020). Additionally, data from a MAGIC population described by Diaz et al. (2019) (manuscript submitted) was used as comparison to the VEF panel regarding parameter optimization for GP. This population was developed from eight elite Mesoamerican founder breeding lines and, therefore, differs from the VEF population in genetic characteristics and germplasm background.

### Field Trials VEF

In total twelve field trials took place in Palmira, Colombia (3°29′′N latitude, 76°21′′W longitude and an altitude of 965 m a.s.l.) and in Darien, Colombia (3°53′′N latitude, 76°31′′W longitude, altitude 1,500 m a.s.l.). The trials were carried out with an alpha-lattice experimental design, with variable replicate numbers and plot sizes (**Supplementary Table 1**). Eight trials in Palmira had three repetitions, two trials in Darien and one trial in Palmira had two repetitions. One trial in Darien was only partially replicated (Dar16C_loP) with 20% of the plots having a second replicate evenly distributed in the field. Field trials were labeled with the location first, Pal for Palmira and Dar for Darien, followed by the year and season, ‘A’ for the first and ‘C’ for the third season, and an abbreviation for the treatment.

#### Field Conditions

Six out of the twelve trials were established under rain fed (to obtain drought stress conditions, see Polania et al. (2017)) and three trials under irrigated conditions in Palmira between 2013 and 2018. Additional three trials were carried out under different levels of P in Darien in one season in 2016. Rain fed trials were carried out during the third trimester of the year, when the precipitation is usually low. These trials were irrigated up to 22 days after sowing, and rain fed only thereafter (see **Supplementary Figure 2** for weather data). Irrigated trials in Palmira were watered during the complete growing season with up to one irrigation event per week, representing 25 mm of rainfall.

The plot sizes were between 2 and 4.4 m^2^, depending on the trial (**Supplementary Table 1**). The field in Darien was divided into three different sections based on the soil P content resulting in low P (loP), medium P (mdP) and high P (hiP) conditions (**Supplementary Table 2**).

#### Crop Management

Standard field practices were applied over the plant growing seasons across trials, including the application of fungicide seed treatment and foliar insecticides when necessary.

#### Weather Data

Compact weather station (Lambrecht meteo GmbH, Göttingen, Germany) equipped with precipitation and temperature sensors was used to collect environmental data. The station is placed at the CIAT station in Palmira about 1 km from the field.

### Phenotyping VEF Panel

The number of days to flowering (DF) was measured from planting to the day when 50% of the plants in the plot had at least one open flower. Days to physiological maturity (DPM) was measured as the number of days from planting until 50% of plants had at least one pod losing its green pigmentation. Seed weight (100SdW, g 100 seeds^−1^) was obtained from weighing 100 seeds. Yield (kg ha^−1^) was calculated based on the plot size and corrected to a moisture content in the seed of 14%.

### Analysis of Phenotypic Data

The spatial arrangement of plots in the field was used to assign row and column coordinates in each trial. The phenotypic data was analyzed using a combined two-stage approach. The first-stage linear mixed model with spatial correction was fitted for each trial separately. This model included random effects for rows, columns and a bilinear polynomial and smoothing spline using the functions ‘SpATS’ and ‘PSANOVA’ of the R package SpATS (v1.0-9) (Rodríguez-Álvarez et al., 2018). In addition, the model incorporated a categorized number of harvested plants per plot as a random effect factor. Observations with extreme residuals were removed, in as many iterations until no residuals beyond ±3 standard deviations were left. Best linear unbiased estimators (BLUEs) and standard errors (SE) were obtained for each line in every trial from this first-stage model. These BLUEs were used for i) calculation of Pearson correlation coefficients to describe the correlation between the different trials, ii) genomic prediction of new lines into an observed environment (i.e. in an observed trial), iii) prediction of new lines into a future season and iv) carrying out GWAS separately for each trial.

A second-stage model was fitted to calculate BLUEs over all trials using the ‘*lm’* function of base R and the emmeans package (Lenth, 2019) with the following equation:

(1) y=trial+G×E+ε

where y is a vector of first-stage BLUEs, trial is a fixed effect factor for each of the twelve trials, G is a vector with each line, and E is a three-level factor indicating the conditions in Darien, the drought and the irrigated trials in Palmira. The residual error is ϵ with ϵ ∼ N(0, σ^2^D(V)), where D(V) is a diagonal matrix with diagonal elements equal to those SE derived by SpATS as described in Möhring and Piepho (2009). The second-stage BLUEs were used to i) obtain overall PAs, ii) to run GWAS over all trials and over environmental conditions separately, and iii) to get overall phenotypic correlations between traits.

### Genotyping by Sequencing (GBS)

DNA was extracted as described by Lobaton et al. (2018) and Perea et al. (2016). In short, leaf tissue from germinated seedlings was frozen with liquid N_2_ and ground to a fine powder with metal balls in a paint shaker. The DNA from this powder was extracted with the Urea buffer-based mini prep extraction protocol. The concentration of the product was estimated by visualization in a 0.8% agarose gel after electrophoresis and thereafter quantified with a NanoDrop 1000. The GBS library was generated using *Ape*KI-based restriction digestion, following the protocol of Elshire et al. (2011). DNA sequencing was performed at the Cornell and HudsonAlpha sequencing facilities on Illumina HiSeq machines. The GBS libraries were sequenced using either single-end or paired-end sequencing with read lengths ranging between 101 and 151 bp.

### SNP Calling and Population Structure

SNP calling was carried out as described by Gil et al. (2019). Briefly, the GBS reads were demultiplexed using NGSEP (v3.3.0) (Tello et al., 2019). Adapters and low quality bases from the raw sequencing data were trimmed using Trimmomatic (v0.36) (Bolger et al., 2014), and the processed reads were aligned to the reference genome of *P. vulgaris* G19833 v2.1. (Schmutz et al., 2014) using Bowtie2 (v2.2.30) with default parameters (Langmead and Salzberg, 2012). The variant calling process was performed using NGSEP following recommended parameters for GBS data (Perea et al., 2016). The merged genotypic matrix was filtered with NGSEP for variants with a genotype quality above 40 for each SNP, minor allele frequency (MAF) above 0.05, a maximum observed heterozygosity of 0.05 per marker, and keeping only biallelic single nucleotide variants. In addition, the variants located in repetitive regions of the reference genome were removed, as reported by Lobaton et al. (2018). The resulting filtered matrix contained about 20% of missing genotype calls over all SNPs after removing markers with less than 270 individuals genotyped per variant. This data was imputed using Beagle v.5.0 (Browning et al., 2018) providing the genetic map reported by Diaz et al. (2019) and setting the effective population size to 100. This matrix was used to assess the population structure in the panel by performing a principal component analysis (PCA) as implemented in GAPIT (Tang et al., 2016). The population structure was also evaluated by calculating a distance matrix and then computing a neighbor-joining tree with NGSEP (v3.3.0).

### Genome-Wide Association Study (GWAS)

GAPIT was used with the recently developed BLINK algorithm to carry out GWAS (Huang et al., 2019). BLINK improves the power to detect true QTL by adding pseudo quantitative trait nucleotides as covariates (Huang et al., 2019). In addition, the first three PCA components were used to correct for population structure. The threshold for significant marker-trait association was defined according to the false discovery rate calculated with the brainwaver R package (Achard, 2012). GWAS was carried out using the first-stage BLUEs obtained from each trial’s SpATS model, and the second-stage BLUEs from Equation (1), obtained for every environmental condition (Palmira-drought, Palmira-irrigated and Darien) and over all trials.

### Genomic Prediction

Predictions were calculated using the BGLR package in R (Pérez and de los Campos, 2014). The number of Markov Chain Monte–Carlo iterations per model run was 10,000 with the first 5,000 as burn-in.

#### Prediction of New Lines

The basic equation to account for additive genetic effects was:

(2) $$y=Z_{1}Gu_{1}+\epsilon$$

where y is a vector of phenotypes, G is the SNP matrix with each marker in one column, u_1_ is the random effect of the SNP markers, where u_1_ ~ *N*(0, I σ_u_^2^). Z_1_ is a design matrix connecting phenotypes and lines and ϵ is the random error, where ϵ ~ *N*(0, I σ_ϵ_^2^). This model is referred to as the marker model. It was used to calculate GEBVs for each trait and trial independently, using first-stage BLUEs; and overall GEBVs for each trait, using second-stage BLUEs as true breeding values (TBVs). Using the SNP matrix results instead of the genomic relationship matrix (GG^T^/p, where p is the number of markers) allows to select SNP markers directly for further breeding purposes (Endelman, 2011).

#### Prediction of New Lines Accounting for G × E

The equation to account for G × E was:

(3) $$y=S_{1}a+E_{1}b+Z_{1}Gu_{1}+Z_{2}Gu_{2}+\epsilon$$

where y is a vector of first-stage BLUEs, a is the fixed effect of the season design matrix S_1_, b is the fixed effect of the environmental design matrix E_1_, u_2_ is the slope of the SNP marker effect, where u_2_ ~ *N*(0, I_E_ ⊗ I σ_u_^2^) and ⊗ denotes the Kronecker product. Z_2_ is a design matrix connecting phenotypes, lines and environments. Z_1_ and u_1_ are defined as described above. I_E_ is the diagonal matrix for the levels of environmental factor E. Different levels for E were constructed:

- The trials in Darien (with different P conditions in the soil), the drought trials in Palmira, and the irrigated trials in Palmira were considered as three distinct environments (Darien, Palmira drought, Palmira irrigated). The resulting model was called Marker * Env model.
- The trials with drought and low P conditions were considered as a stressed environment. The remaining trials were considered as a non-stress environment. The resulting model was called Marker * Stress model.

#### Prediction of New Lines Accounting for G × E and QTL Effects

Significant SNP markers from the GWAS using second-stage BLUEs for all trials were included additionally in the Marker * Env model as fixed effects. This model was named QTL model. The equation to account for multiple environments and QTL effects was:

(4) $$y=S_{1}a+E_{1}b+Q_{1}c+Z_{1}Gu_{1}+Z_{2}Gu_{2}+\epsilon$$

where y is a vector of first-stage BLUEs, c is the vector of fixed effects for the significant QTL based on the Bonferroni corrected *p* value at 0.05 significance level. Hereby, QTL analysis was carried out on adjusted genotypic values as described in the section GWAS without adding principal components. Q_1_ is the design matrix for the identified QTL.

### Parameter Optimization for Genomic Prediction

In order to optimize PAs, three different settings were tested using i) different modeling algorithms, ii) different amounts of SNP markers, iii) different proportions of training and validation sets. The overall second-stage BLUEs obtained from Equation (1) and the SNP matrix of the VEF lines were used for predictions according to Equation (2). In addition, the phenotypic and genotypic data from the MAGIC population of common bean was included in this test as a contrasting population.

The first setting tested different priors for parametric linear regressions on the genetic markers including i) a Gaussian (Bayesian ridge regression), ii) scaled-t (BayesA) or iii) double-exponential priors (Bayesian Lasso); two-component mixtures with a point of mass at zero with either iv) a Gaussian (BayesC) or v) a scaled-t slab (BayesB). In addition, Bayesian Reproducing Kernel Hilbert Spaces (RKHS) regressions were tested fitting vi) a GBLUP model providing a kinship matrix and vii) a single Gaussian kernel RKHS model with fixed bandwidth parameter. The second setting tested a reduced set of markers that was chosen either randomly or based on a MAF and linkage disequilibrium (LD) criteria. The marker filtering based on LD was done using the command ‘indep-pairwise’ of PLINK (v1.90b6.9) pruning markers above the LD threshold in 50 kbp windows (Chang et al., 2015). The step-size parameter was 5 kbp and the LD *r*² thresholds were 0.3, 0.5, 0.7, 0.9 and 0.95. The last setting tested for variable proportions of the training population, ranging from 5 to 95%. All these settings were tested with 100 fold random cross-validation for each trait separately.

### Modeling Optimization for Genomic Prediction

For modeling optimization, Bayesian ridge regression was chosen to solve the linear model. Predictions were done using randomly 70% of the lines as the training set and 30% as the validation set. The validation set was either from the same season as the training set (new line in observed season) or it was different from the seasons in the training set (new lines in a future season). In the latter case, 30% of the lines were taken as the validation set and thus were removed from all the remaining seasons in the training set. The PA is expressed as Pearson correlation coefficients between GEBVs and TBVs. The cross validation was repeated 100 times.

#### Cross-Validation of New Lines in the Observed Season

Predictions of new lines were done using Equation (2) within each trial separately and overall trials based on first- and second-stage BLUEs, respectively. New lines means that these individuals had no observation in the training set.

#### Cross-Validation of New Lines in Future Season for Model Comparison

New lines were predicted into a future season based on the remaining lines in the remaining seasons. This means that the new individuals and the predicted future season had no observations in the training set. All different models described were calculated using Equations (3) and (4).

#### Cross-Validation of New Lines in Future Seasons While Cumulatively Adding Data

Predictions of new lines in future seasons were based on trials that were chronologically done before the predicted trial. Additionally, future seasons described seasons whose observations were not used in the training set, i.e., the future season was predicted based on all the remaining seasons (regardless of the chronological order). The first scenario aims to mimic a breeding program where data accumulates over the seasons that are used to predict new lines in future seasons. The second aims to compare the prediction for new lines in future, unobserved seasons when the training set has always the same amount of seasons, i.e., all but the one season which is to be predicted. The Marker * Env model described in Equation (3) was used for these predictions. The 100-fold cross-validation was done as described above. Second-stage BLUEs of phenotypes in the training set were calculated using Equation (1) in order to determine the phenotypic correlations with the predicted season.

### Heritability

Genomic heritability was calculated using post mean genetic variance according to De los Campos et al. (2015) and Lehermeier et al. (2017b). Genomic heritability takes the variance explained by the genomic markers as genetic variance instead of the variance explained by the lines (as in the conventional calculation of heritability) (De los Campos et al., 2015). The extended approach of Lehermeier et al. (2017a) averages the variance of the GEBVs within the thinned Markov chain Monte Carlo. The number of iterations was 10,000 with the first 5,000 as burn-in without partition of training and validation set. The remaining 5,000 iterations were thinned by factor 10. Then the genetic variance (variance of the GEBVs) and error variance was divided by the genetic variance in all of these iterations and averaged.

The broad-sense heritability was calculated using the function ‘getHeritability’ provided in the SpATS package, which uses the nominal and effective dimensions of the genotypic term when it is fitted as a random effects factor (Oakey et al., 2006; Rodríguez-Álvarez et al., 2018). Broad-sense heritability was calculated for each trial separately and for all trials together.

## Results

### Phenotypic Evaluation of Field Trials

**Figure 1:**
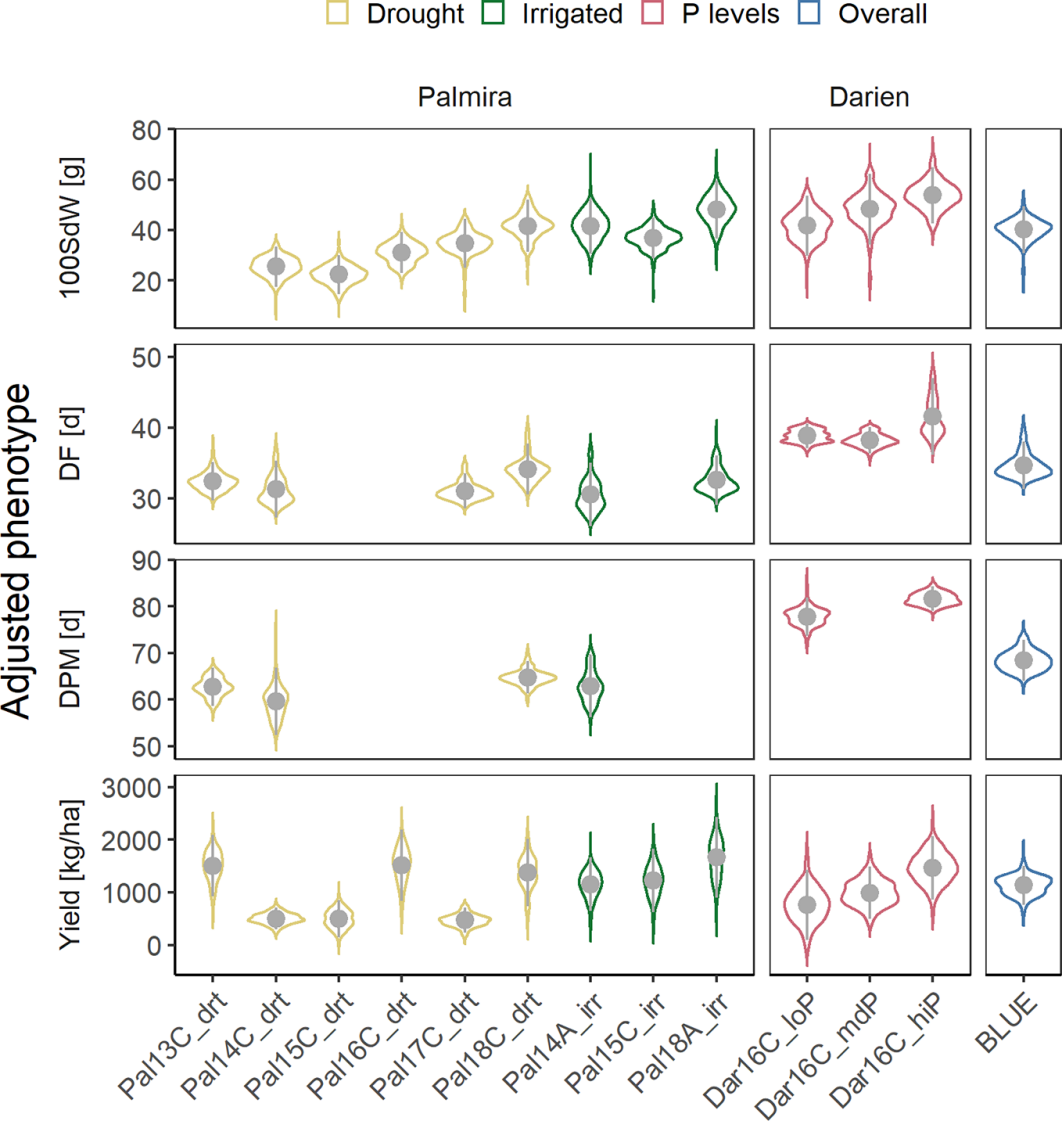
Phenotypic variability of 100 seed weight (100SdW), days to flowering (DF), days to physiological maturity (DPM), and seed yield evaluated in up to twelve field trials. The trials were carried out between 2013, first planting season ‘A’ and 2018 third season ‘C’ under drought, irrigated and different phosphorus (P) conditions in Darien (Dar) and Palmira (Pal), Colombia. In total, 481 common bean lines were evaluated (between 156 and 345 lines per trial). Best linear unbiased estimators (BLUEs) were obtained for each trait and trial, adjusting for spatial effects in the field in a first-stage analysis. In addition, weighted BLUEs were calculated over all trials in a second-stage analysis.

Twelve field trials were carried out in two locations to evaluate agronomic traits, under irrigated and drought conditions in Palmira and under different P conditions in Darien. Between 156 and 345 lines were evaluated in each trial (**Supplementary Figure 1**). The measured traits, 100SdW, DF, DPM and seed yield, showed strong season-to-season variation (**Figure 1**). The variation between seasons and locations was associated with the different climatic conditions (**Supplementary Figure 2**). Particularly, DF and DPM were delayed in the higher altitude location Darien compared to Palmira (**Figure 1**). The 100SdW was generally lower under rainfed conditions and the three lowest yielding trials were observed under rain fed conditions, indicating drought stress. However, total precipitation during a trial showed only a weak correlation with yield, suggesting significant factors other than drought constraining yield (**Supplementary Figure 3**).

Phenotypic correlations between trials were positive for all traits (**Supplementary Figure 4**). Most correlations between trials on the same location were highly significant with higher coefficients in DF (between 0.25 and 0.87) than yield (between 0.22 and 0.72). These values were lower comparing trials from different locations for DF (between 0.13 and 0.65) or yield (0.2 and 0.36). Broad-sense heritabilities were high for DF (0.55 to 0.97), DPM (0.66 to 0.93) and 100SdW (0.81 to 0.95) and lower for yield (0.45 to 0.85). The trial with the lowest yield heritability in Palmira, Pal14C_drt, was affected by a virus infection, leading to increased variation and lower phenotypic correlations with other trials. There was no clear pattern of trait correlations between drought and irrigated conditions (**Supplementary Figure 4**). The correlation of DF and DPM with seed yield over all trials was slightly negative (**Supplementary Figure 5**). In summary, the heritabilities were rather consistent among traits and no extreme G × E effects were observed across the twelve trials.

### Genotypic Results

**Figure 2:**
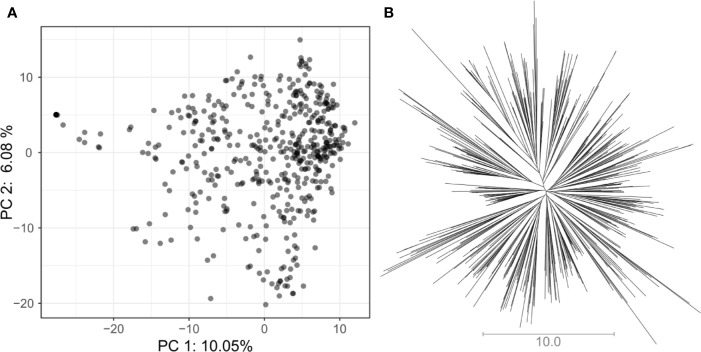
Assessment of population structure for the VEF population including 481 common bean lines with 5,820 SNP markers: **(A)** The principal component analysis shows the location of each genotype defined by the eigenvectors of the first and second principal components. **(B)** The unrooted neighbor-joining tree indicates the absence of a clear differentiation pattern between lines. The length of the lines in the tree shows the simple matching distance.

In total, 5,820 high-quality SNP markers were called in the 487 lines of the VEF panel. The missing genotype calls were set to 20% before imputation. After removing markers with identical calls within the population, 4,962 markers remained. A low population structure was visible in the VEF panel (**Figure 2**). The first two PCA axes explained 16% of the variance in the SNP data. A few samples in **Figure 2A** were located slightly distant from the main cloud of points, which likely contain a higher level of admixture with the Mesoamerican genepool. However, no defined clusters were visible in the distribution of samples in the two-dimensional space. Similarly, the neighbor-joining tree shows small clusters with no clear pattern of group differentiation (**Figure 2B**). Taken together, these results indicate a moderately low population structure as expected from a panel of breeding lines.

### QTL Mapping

**Figure 3:**
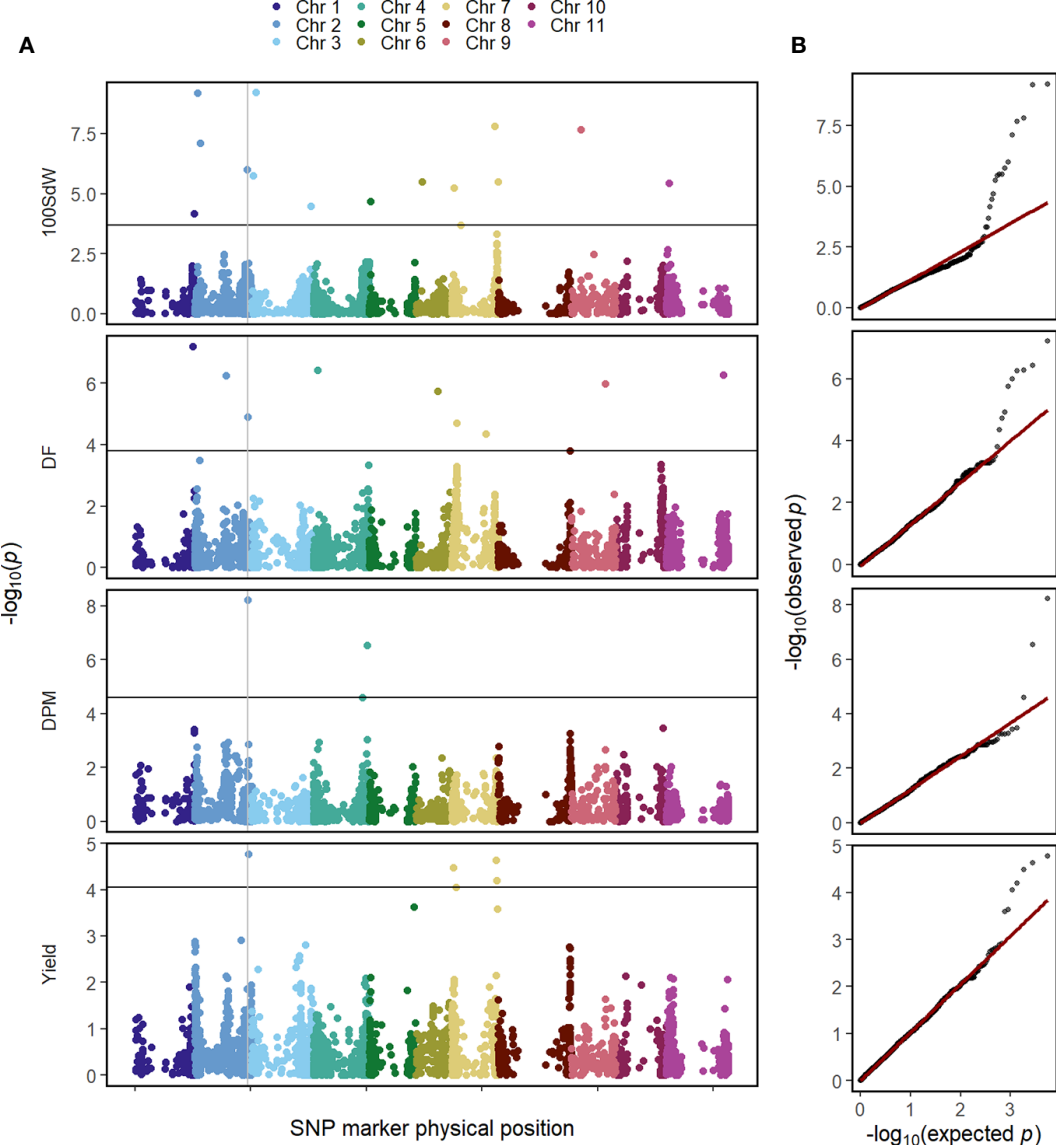
Genome-wide association study for in total 481 common bean lines using second-stage best linear unbiased estimators (BLUEs) across twelve trials for the traits 100 seed weight (100SdW), days to flowering (DF), days to physiological maturity (DPM), and seed yield. **(A)** The Manhattan plots show the significance of associations of every SNP marker for each trait calculated using the BLINK algorithm, implemented in GAPIT. The vertical line indicates a common QTL for DF, DPM and seed yield. The horizontal line shows the false discovery rate (*p* = 0.05) to identify significant associations. **(B)** Q–Q plots show the distribution of the negative logarithm of expected and observed *p* values.

The GWAS was conducted on the whole population in order to identify QTL in this elite breeding panel within and across trials and conditions. Several QTL were identified for all evaluated traits when using second-stage BLUEs over all twelve trials (**Figure 3**). Major QTL were identified at the end of Chr 1 at 49.7 megabase pair (Mbp) and the beginning of Chr 2 at 4.6 Mbp for DF and 100SdW, respectively. Interestingly, another QTL at Chr 2 at 46.4 Mbp was found repeatedly for DF, DPM and seed yield (**Supplementary Table 3**). Furthermore, an additional yield QTL to Chr 7 was mapped between 38 and 39.5 Mbp.

In the following, GWAS were conducted using second-stage BLUEs for the different environmental conditions (**Supplementary Figure 6**). One QTL at the beginning of Chr 2 between 1.4 and 4.7 Mbp was identified for 10SdW and DF under all three environmental conditions. The QTL at the end of Chr 2 was detected again for seed yield under all three environmental conditions between 46.2 and 46.6 Mbp. Regarding DF, a QTL on Chr 1 was detected under irrigated conditions in Palmira and in the location Darien between 42.5 and 44.6 Mbp, while under drought conditions in Palmira a major QTL on Chr 9 at 29.9 Mbp was identified.

To investigate QTL stability among the twelve trial conditions, GWAS was conducted for each trial independently using first-stage BLUEs (**Supplementary Figure 7**). The detected QTL were rather inconsistent among the different trials: the most stable QTL was detected for DF on Chr 2 at around 4.2 Mbp in five out of the twelve trials.

### Optimization of Genomic Prediction Parameters

To identify optimal GP settings, the effect of several parameters on the PA were evaluated in the Andean VEF panel and compared to the Mesoamerican MAGIC population (Diaz et al., 2019). The MAGIC population was developed by intercrossing eight Mesoamerican breeding lines, resulting in a lower population structure (**Supplementary Figure 8**). For the first test, different parametric models were evaluated for the four traits. The tested algorithms generally performed very similar (**Supplementary Figure 9**). The RKHS algorithm slightly outperformed the other algorithms in several traits, apart from DF in the MAGIC population. However, it does not return SNP marker effects. Therefore, for the remaining analyses, Bayesian ridge regression was chosen.

**Figure 4:**
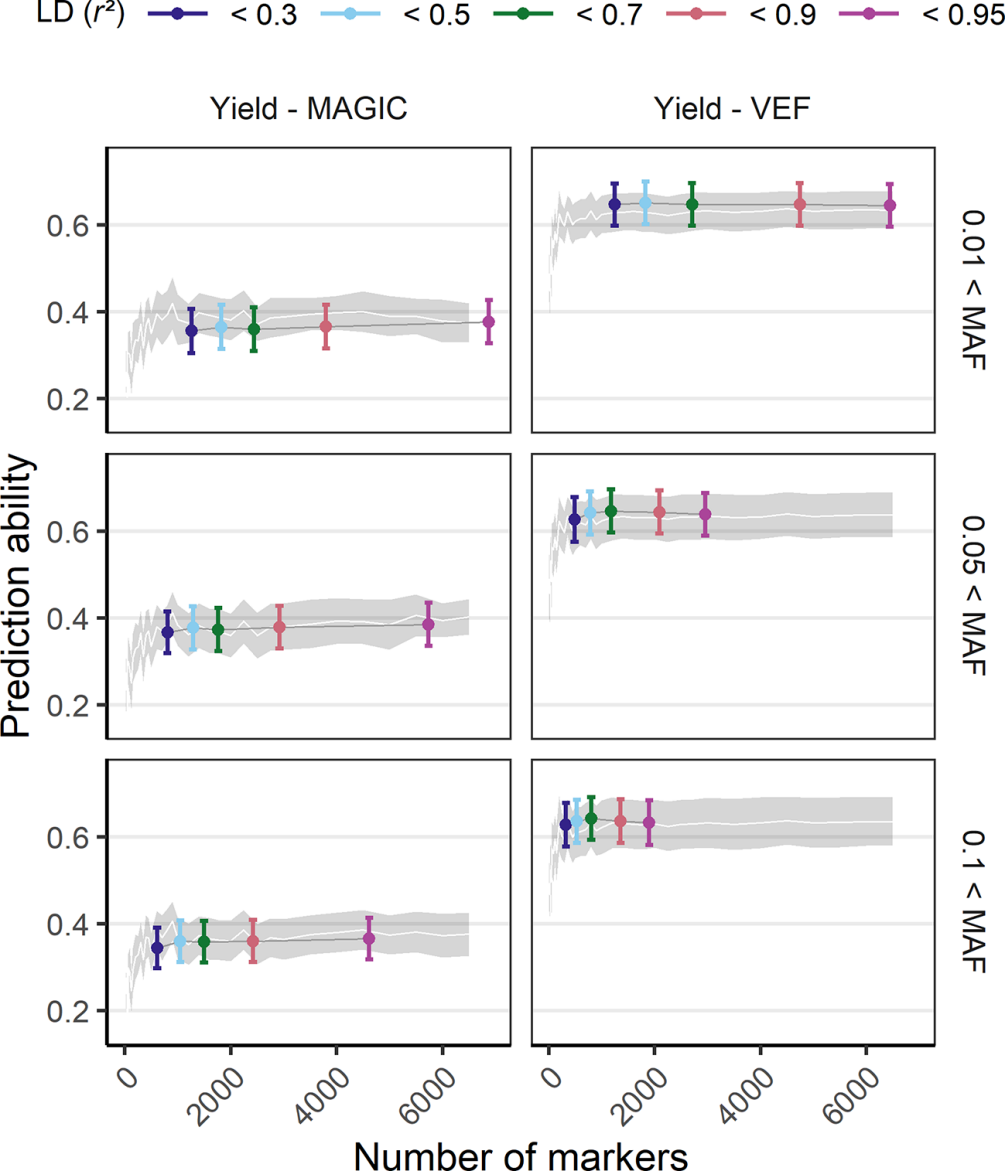
Genomic prediction abilities of seed yield in response to the number of utilized markers evaluated in the VEF and the MAGIC populations. The markers used for prediction were chosen either randomly (the white line and its gray stripe show the average prediction ability and its corresponding standard deviation) or based on LD and MAF parameters (colored ranges, the middle point and its error bar represent the average prediction ability and its standard deviation). The distribution of values in this plot corresponds to 100-fold cross validations with 70:30 training:validation population partitioning.

The effect of marker density on PA was tested by a reduction of the original number of markers either randomly, based on LD or based on MAF (**Figure 4**, **Supplementary** **Figure 1** **0**). Models using marker sets with a higher MAF threshold (0.1) had a decreased maximum PA while the marker sets with the smallest MAF filter (0.01) showed best performance. Reducing markers based on LD showed that the number of markers could be reduced to 1,000 without losing PA. In the VEF panel, marker reduction based on LD was more successful compared to random marker reduction, increasing PAs up to 4%. However, the same strategy produced rather unfavorable effects on PAs (up to −2%) for the MAGIC population. In general, there was no clear detrimental effect of larger marker numbers on PAs, even if the markers were of lower quality. The more markers were used the better were the PAs for all traits.

Partitioning between training and validation set showed an optimum at around 70:30% (**Supplementary Figure 11**). A training set of less than 30% strongly reduces PA due to an insufficiently sized training set that results in overfitting of the model. Similarly, a training set >80% leads to large variation between cross validations due to an excessively small validation set that is not robust when some outliers are present. Interestingly, the larger MAGIC population showed a similar behavior even though it would be expected that a larger number of individuals would reach a plateau in PA with a smaller training set percentage.

### Genomic Prediction Abilities for Agronomic Traits

**Figure 5:**
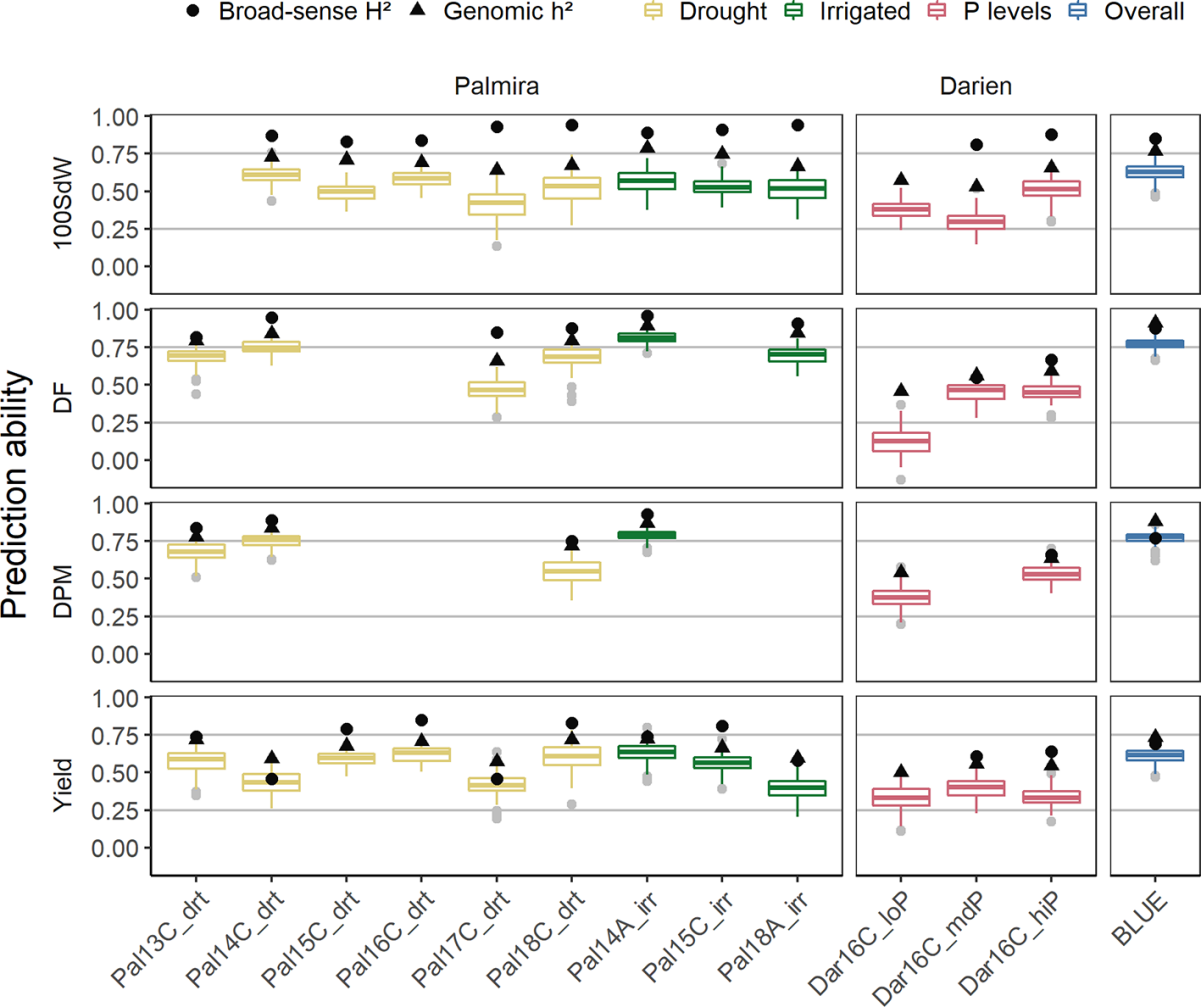
Genomic prediction abilities evaluated separately for each of the twelve trials as well as over all seasons in a total of 481 lines. The 100 seed weight (100SdW), days to flowering (DF), days to physiological maturity (DPM), and seed yield were evaluated between 2013 and 2018 in season ‘A’ or ‘C’, under drought, irrigated and different soil phosphorus (P) conditions in Darien (Dar) and Palmira (Pal), Colombia. The model predictions were based on 4,962 SNP markers using Bayesian ridge regression. In the overall predictions, new lines were predicted based on the second-stage best linear unbiased estimators (BLUEs) obtained from all the trials. Black dots and triangles indicate broad-sense and genomic heritability, respectively.

Genomic PAs for each trial and across all trials were calculated. PAs differed between trials and traits and followed the broad-sense and genomic heritability (**Figure 5**). PAs within trials reached in general 50 to 80% of the genomic heritability, both for complex traits as yield and more simple traits like DF. Average PAs for yield ranged between 0.25 and 0.6, whereas the remaining traits reached higher values up to 0.8. The missing heritability, which is the difference between the broad-sense and genomic heritability, seemed to be highest in the 100SdW trait.

PAs were similar between drought and irrigated conditions in Palmira. Beyond differences in heritabilities, there was no strong trend indicating that certain seasons show better PAs than others. The trials in Darien, which only had two replicates, had in general lower PAs. The low P trial resulted in similar PAs as the higher P trials, regardless that this trial was only partially replicated. Because of the few partial replicates, the broad-sense heritability was not calculated for this trial. In summary, the PAs were dependent on the trait heritability of each trial.

Across all seasons, PAs for new lines based on the BLUEs reached 0.8 for DPM and 0.6 for seed yield (**Figure 5**). The SNP marker effects obtained from the BRR model over all seasons are shown in **Supplementary Figure 12**.

### Prediction Abilities Between Trials—Prediction Model Improvement With G × E and QTL Co-Factors

**Figure 6:**
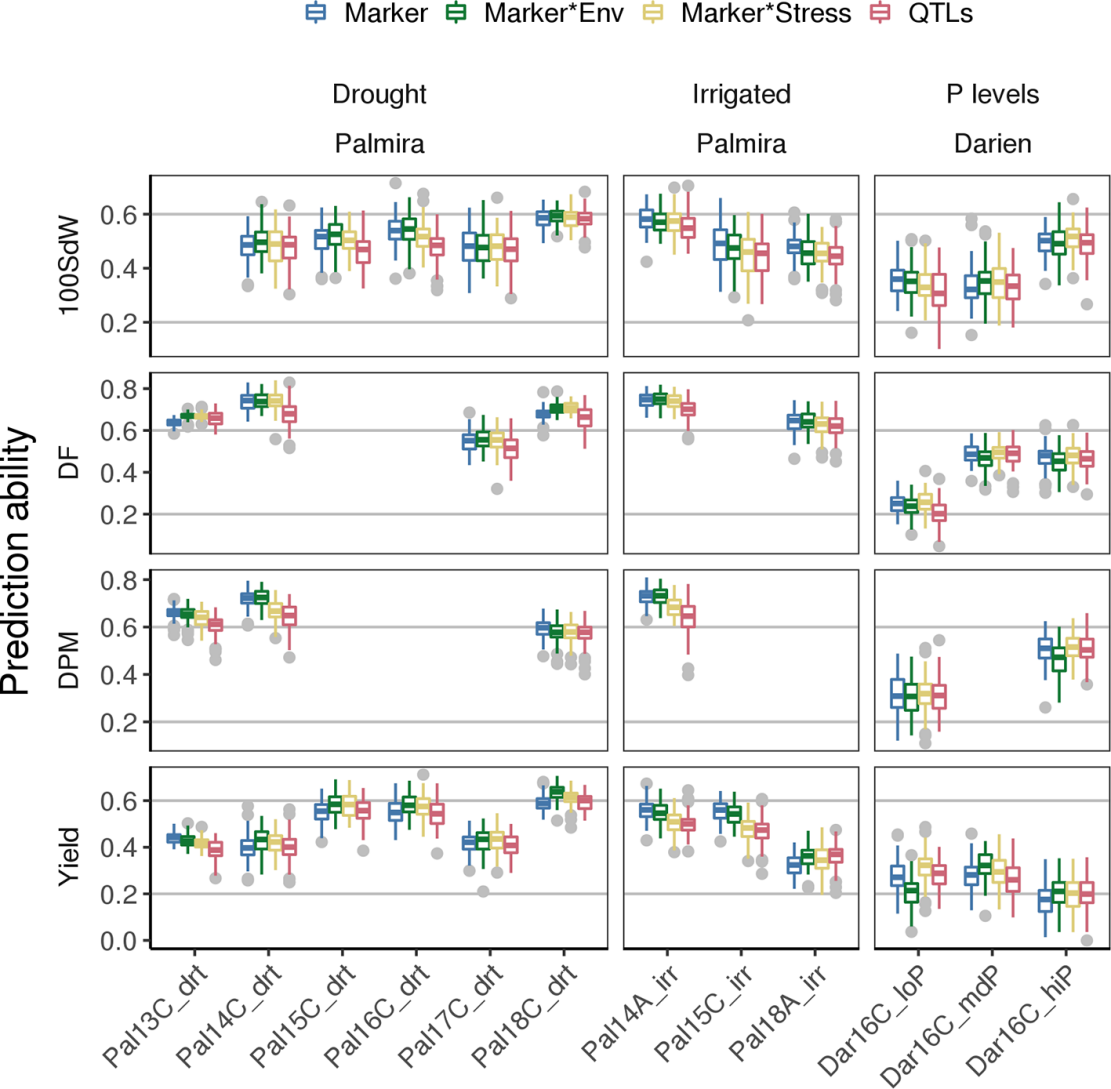
Genomic prediction abilities for new lines in new seasons compared for different models using 100 fold cross validation. Different genotype × environment interactions were considered to improve the basic SNP marker model among different traits: Modeling the effects of drought and irrigated conditions in Palmira and the location effect of Palmira separately (Marker * Env model), the stress effect for drought and low P conditions (Marker * Stress model) or the Marker * Env with fixed QTL effects (QTL model). The 100 seed weight (100SdW), days to flowering (DF), days to physiological maturity (DPM), and seed yield were evaluated in up to twelve trials. The trials were carried out between 2013 first planting season ‘A’ and 2018 third season ‘C’ under drought, irrigated and different phosphorus (P) conditions in Darien (Dar) and Palmira (Pal), Colombia.

PAs for new lines in future seasons (whose data was not used to generate the prediction model) depend strongly on the environmental conditions present in such future seasons. The modeling of G **×** E accounts for specific responses of lines to environmental conditions. This could improve PAs for new lines in a new season when a trial with similar environmental conditions is present in the training set. Including G **×** E factors in the GP model improved PAs especially under drought conditions (**Figure 6**). For example, the Marker * Env model increased PAs for DF and seed yield in most of the drought trials. Particularly, PAs for yield increased between 3 and 10% based on the mean of the 100-fold cross-validation, except in Pal13C_drt where PAs decreased slightly. Interestingly, in the low P trial, prediction for seed yield could be improved by 22% when including stress interaction from the Marker * Stress model. This model was trained on all the remaining trials including the genotypic interaction under drought conditions. It indicates that adding drought specific effects in the model improves PA for low P conditions. The variance explained by the G **×** E terms reached 24% in yield when using lines as in Equation (1) (**Supplementary Figure 13A**). It was below 5% for all traits and both tested models when lines were represented by the molecular markers as in Equation (3) (**Supplementary Figure 13B**). As a second potential model improvement, SNP markers associated with QTL were added as fixed effects to the genomic model (QTL model). However, this did not result in improved PAs and the selected QTL on the training set varied often (**Supplementary Figure 14**). In summary, an increase of PAs for future seasons was observed for trials under abiotic stress conditions by taking into account GxE but not QTL effects.

### Prediction Ability With Increasing Data—Building a Cumulative Prediction Model Over Consecutive Years

**Figure 7:**
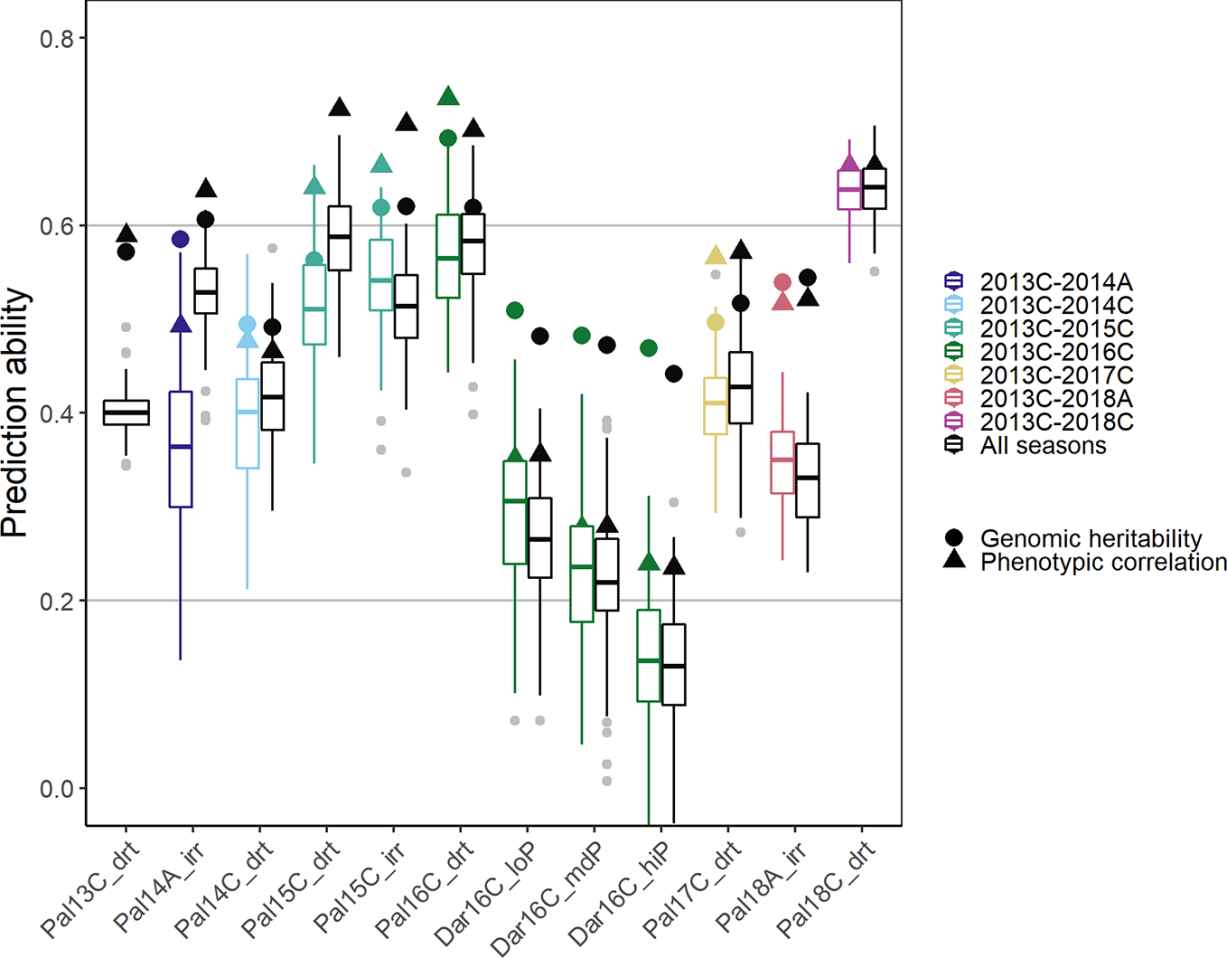
Genomic prediction abilities for seed yield in a breeding program using chronological data accumulated up to the predicted season (colored boxplots), and data over all seasons (black boxplots) to predict new lines in new seasons using 100 fold cross validation. In total 4,962 markers were fitted using Bayesian ridge regression and the Marker * Env model (see *Material and Methods*). In total, twelve trials between 2013 first planting season ‘A’ and 2018 third season ‘C’ under drought, irrigated and different phosphorus conditions in Darien (Dar) and Palmira (Pal), Colombia were evaluated.

In order to simulate processes in a breeding program, new lines were predicted in a new season based on the training set of accumulated data from prior seasons (**Figure 7**). A breeding program could make use of collected data to develop prediction models over several years, i.e., the training set consists of all data acquired cumulatively up to the new predicted season. As a comparison for optimal prediction of a particular season, we used the investigated season as the validation set and all remaining seasons as training data (including those before and after the analyzed season). In the cumulative sequence, the first trials were predicted with lower accuracy. The PA increased in general when more data was accumulated over the years reaching values close to the genomic heritability after around three seasons (**Figure 7**, colored boxplots). The trials in the new location Darien with no previous data available were predicted with low accuracy. In these trials, the phenotypic correlation of the new trial with the training set was significantly smaller than the genomic heritability indicating insufficient training data for this location and specific G × E effects. The more data was available from a similar environment the better were the genotypic predictions in a certain season. In consequence, PAs were generally higher when using all the remaining data instead of only the preceding data (**Figure 7**, black boxplots). The phenotypic correlation between the training set and a future season as validation set constitutes the limit for the PA. This represents the accuracy of a breeder’s ability to predict the outcome of a future trial without genotypic information. With more data available, predictions reached accuracies close to phenotypic correlation.

## Discussion

We carried out the most extensive analysis of GP in common bean so far using data of elite breeding lines of the CIAT breeding program. PAs of four traits under different environments increased with more data available and reached usually 50–80% of the genomic heritability. Predictions differed between traits and reached best accuracies in DF (**Figure 5**). The genetic architecture of DF is comparatively simple and has been modelled before (Bhakta et al., 2017; Wallach et al., 2018). However, also complex traits such as yield reached good PAs, e.g. between 0.3 and 0.6 in our study. Barili et al. (2018) achieved lower PAs for grain yield in common bean, however, based on a much smaller dataset containing 80 lines. In chickpea, the PA for seed yield, DF, DPM and 100SdW were similar as in our study, i.e. about 0.5 for seed yield predicting new lines in observed trials (Roorkiwal et al., 2016). These results show that genomic prediction of simple and complex traits is promising for implementation in the bean breeding program.

### Prediction of New Lines Across Environments

Predictions improve with more available data, which increases trait heritability and allows to model G × E (Spindel and McCouch, 2016). About 17% better accuracy was reported for maize yield over twelve years when G × E on a big range of 58 environments were included (Acosta-Pech et al., 2017). Another study found an increase in PA of 11% for height in barley when G × E was accounted for (Oakey et al., 2016). In our dataset, the modeling of G × E increased the PAs of new lines across seasons up to 10% under drought conditions (**Figure 6**). Similarly, in chickpea, G × E did slightly improve predictions for agronomic traits in future seasons (Roorkiwal et al., 2018). In a historical wheat dataset G × E did not improve PA (Dawson et al., 2013). PAs generally increased when more data was available in the training set (**Figure 7**). PAs came close to the phenotypic correlations between trials when all twelve trials were considered as the training set. Previous reports suggested working with less replicates in more environments to account better for G × E, especially in the early selection process (Cooper et al., 2014; Bustos-Korts et al., 2016). Similarly, PAs of the low P trial in Darien with only partial replicates were comparable to the higher P treatments with two replicates. Phosphorus and drought tolerance are genetically linked (Beebe et al., 2008). Thus, these traits can potentially contribute to predicting each other. However, these results need validation with more locations to confirm that the G × E are indeed similar. Including G × E *via* genotypic responses to, e.g., heat or precipitation, could be a promising step in order to further improve PAs (Heslot et al., 2014; Millet et al., 2019). In conclusion, a continuous prediction model over the years provided increasing PAs suitable for breeding applications. Even though the modelled effect of G × E was minor, it allows more accurate selection of superior individuals for specific environmental conditions.

### QTL Mapping and Genomic Prediction

Molecular markers associated with major QTL for different traits are already developed and used in the CIAT breeding program (Diaz et al., 2018; Gil et al., 2019). In the current study, QTL for DF were mapped to Chr 1 between 42.5 and 49.7 Mbp (**Figure 3**). Within this region, the *PvTFL1y* flowering gene was identified on Chr 1 at 44,855,370 bp (Kwak et al., 2012). *PvTFL1y* was reported to explain 66% in vegetative growth and 19% for the rate of plant production (González et al., 2016). Associations close to this gene were also found in another Andean diversity panel (Cichy et al., 2015). The identified QTL for DF under drought conditions on Chr 9 at about 29 Mbp did not match the position reported in earlier studies (at about 14 Mbp) (de Campos et al., 2011). In this study, GWAS was not successful in identifying major QTLs, which were stable over many trials (**Supplementary Figure 7**). This was expected for complex traits, which show strong influence by G × E. Therefore, e.g., the QTL for seed yield found in a diverse germplasm set of Kamfwa et al. (2015) could not be confirmed in our study. In contrast, new lines across seasons were well predicted using GP models. Therefore, selection of single markers does not appear promising for complex traits, whereas GP provides good predictions of phenotypic data based on all available genetic markers.

GP models have been reported to improve PA when significant GWAS-identified markers were incorporated as fixed effects (Bian and Holland, 2017). This strategy works well when the traits are highly heritable and QTL with major effects are detectable (Bian and Holland, 2017; Sarinelli et al., 2019). However, Rice and Lipka (2019) reported that the inclusion of fixed effect QTL negatively affected GP model accuracy. In our panel, no improvement was observed probably because the QTL were not stable over the different trials (**Supplementary Figure 7**).

### Parameters and Algorithms

GPs can be optimized regarding training population and algorithms. In our study, the training population size was optimal when using between 50 and 80% of the population. In general, the larger the training population the greater the prediction potential (Spindel and McCouch, 2016; Sarinelli et al., 2019). Overfitting by using too many markers in the model was not observed in our study. The PA generally saturated after around 1,000 markers, adding more markers did not negatively affect PA (**Figure 4**, **Supplementary Figure 10**). Regarding the different algorithms, most performed similarly as shown in previous studies (Roorkiwal et al., 2016; Bellot et al., 2018). In chickpea, the same traits as in our study were similarly predicted with 3,000 markers using Bayesian priors and random forest (Roorkiwal et al., 2016). Other studies found that different algorithms including deep neural networks performed differently based on the trait (Blondel et al., 2015; Azodi et al., 2019). In such cases, the combination of results predicted by different algorithms (ensemble predictions) may maximize the prediction performance (Azodi et al., 2019). Based on our study, RKHS would be suggested for selection purposes as it is fast and performs well, while Bayesian ridge regression is suggested if marker effect values are desired. Based on the observation that more markers and different models did not increase PA, we conclude that precise and reliable phenotypic data is currently the principal bottleneck of GP, which is tightly linked to experimental design settings like trial size, number of replicates or trial locations.

## Conclusion

GP was evaluated in common bean using twelve field trials with elite Andean breeding lines, testing several models and parameters to identify the optimal model settings. New lines were predicted with PAs of up to 0.6 for yield over all environments and trials, which is close to the estimated heritability. This suggests GP as a powerful tool for selection in breeding programs. Genomic selection can be employed to increase genetic gain through early-generation selection or by replacing costly and time-consuming phenotyping. Based on the marker effects, genomic mating strategies can be further developed to select best possible parents for new crosses (Lehermeier et al., 2017a; Allier et al., 2019). The inclusion of G × E interactions allows specifically selecting for stress tolerant lines to maintain food security under changing climatic conditions.

## Data Availability Statement

The SNP marker matrix, the raw and modeled phenotypic data, as well as the weather data used in this study are available for download at Harvard Dataverse: https://doi.org/10.7910/DVN/XCD67U.

## Author Contributions

BR and BS conceived the study. AP-B, HB and VM conducted the experiments and provided the phenotypic data. BK, DA-S and JA performed modeling, data analysis and interpretation. JH and DA-S prepared the genotypic data. BR and BS assisted in the experimental setup and data analysis. BK drafted the manuscript, which was improved by DA-S, BS and BR. All authors contributed to the article and approved the submitted version.

## Funding

The work was funded by Tropical Legumes III—Improving Livelihoods for Smallholder Farmers: Enhanced Grain Legume Productivity and Production in Sub-Saharan Africa and South Asia (OPP1114827), and by the AVISA—Accelerated varietal improvement and seed delivery of legumes and cereals in Africa (OPP1198373) projects funded by the Bill & Melinda Gates Foundation.

We would like to thank USAID for their contributions through the CGIAR Research Program on Grain Legumes and Dryland Cereals.

The authors would like to thank the COOP Research Program on “Sustainability in Food Value Chains” of the ETH Zürich World Food System Center and the ETH Zurich Foundation for supporting this project. The COOP Research Program is supported by the COOP Sustainability Fund.

## Conflict of Interest

The authors declare that the research was conducted in the absence of any commercial or financial relationships that could be construed as a potential conflict of interest.
