# Supplementary material for: Genomic Prediction of Agronomic Traits in Common Bean (Phaseolus vulgaris L.) Under Environmental Stress
Source: Front Plant Sci. 2020 Jul 7;11:1001. doi: 10.3389/fpls.2020.01001 (PMC7381332; doi:10.3389/fpls.2020.01001)
Supplement: Supplementary file 1 [file DataSheet_1.pdf]

Supplementary Material

1 Supplementary figures

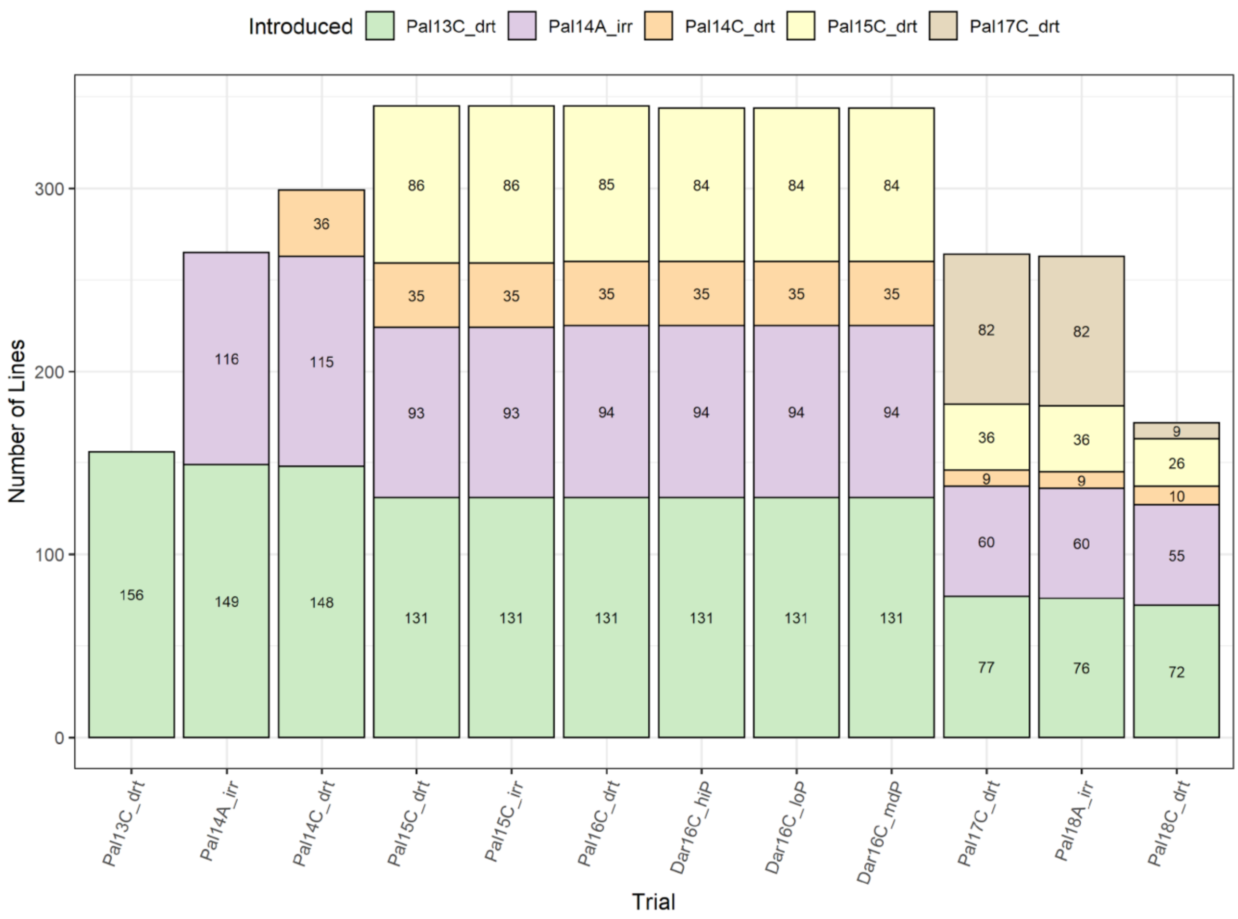

Supplementary Figure 1 Genotype composition across trials of the VEF panel. Evaluations were carried out in Palmira (Pal) or Darien (Dar), during season 'A' or 'C' from 2013 to 2018. Each color represents a new set of lines that was introduced to the panel in the corresponding season.

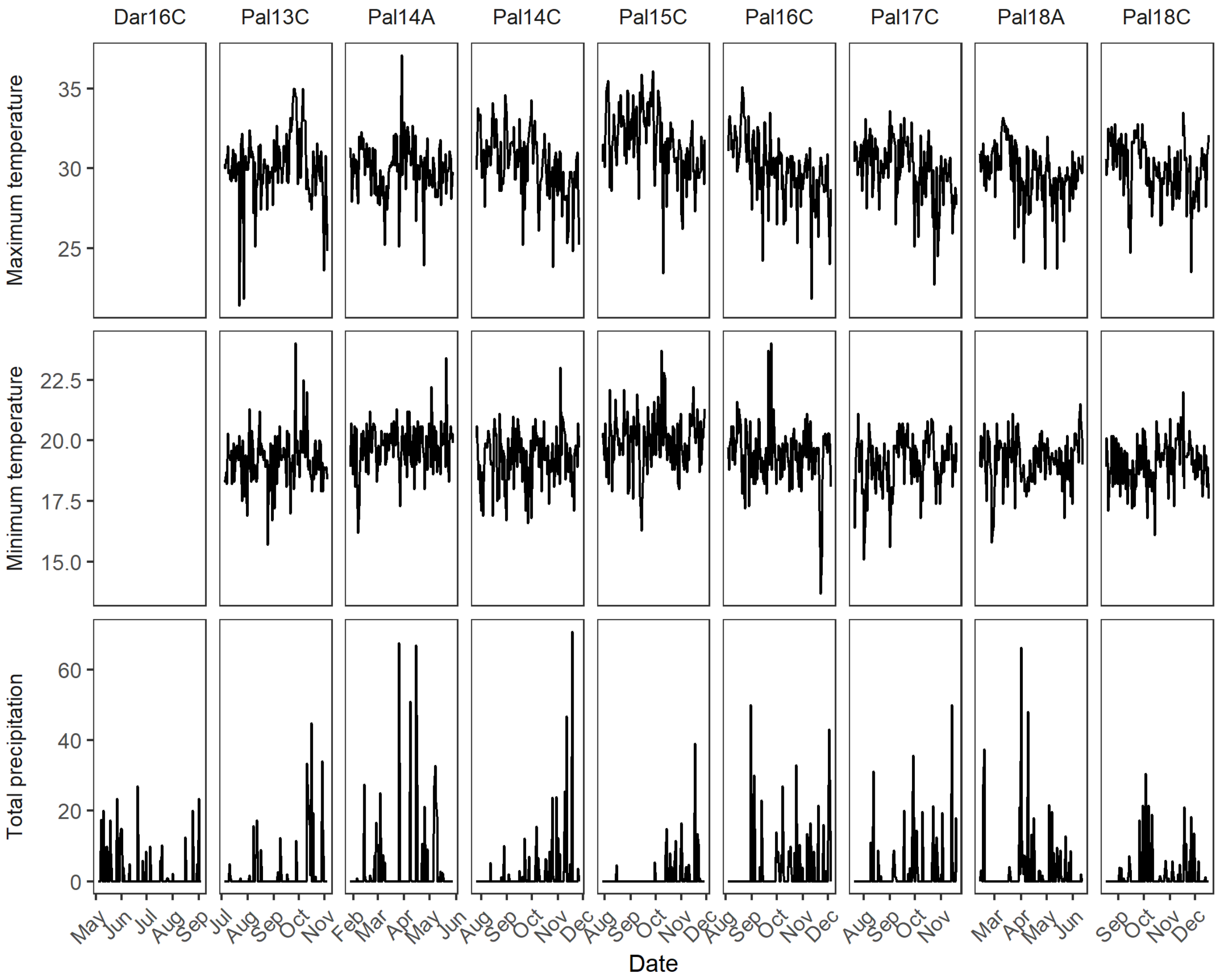

Supplementary Figure 2 Weather data for nine seasons between 2013 first planting season 'A' and 2018 third season 'C' in Darien (Dar) and Palmira (Pal), Colombia. Temperatures are expressed in Celsius degrees and the total precipitation in mm.

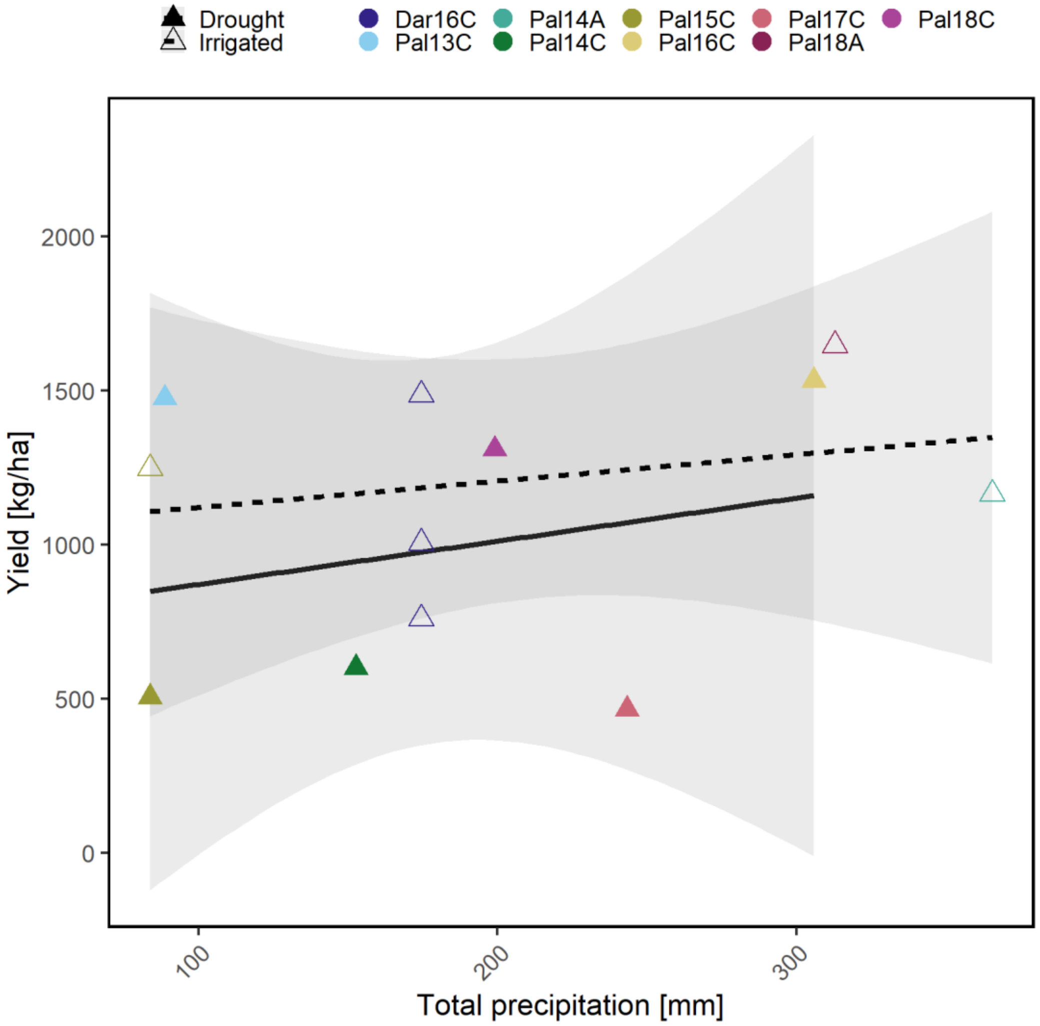

Supplementary Figure 3 Accumulated precipitation from four weeks after sowing until harvest plotted against yield data for twelve trials. The trials were carried out over nine seasons between 2013 first planting season 'A' and 2018 third season 'C' in Darien (Dar) and Palmira (Pal), Colombia. All trials were irrigated until 22 days after sowing. Afterwards, the drought trials were rainfed only, while the irrigated trials were watered up to once a week.

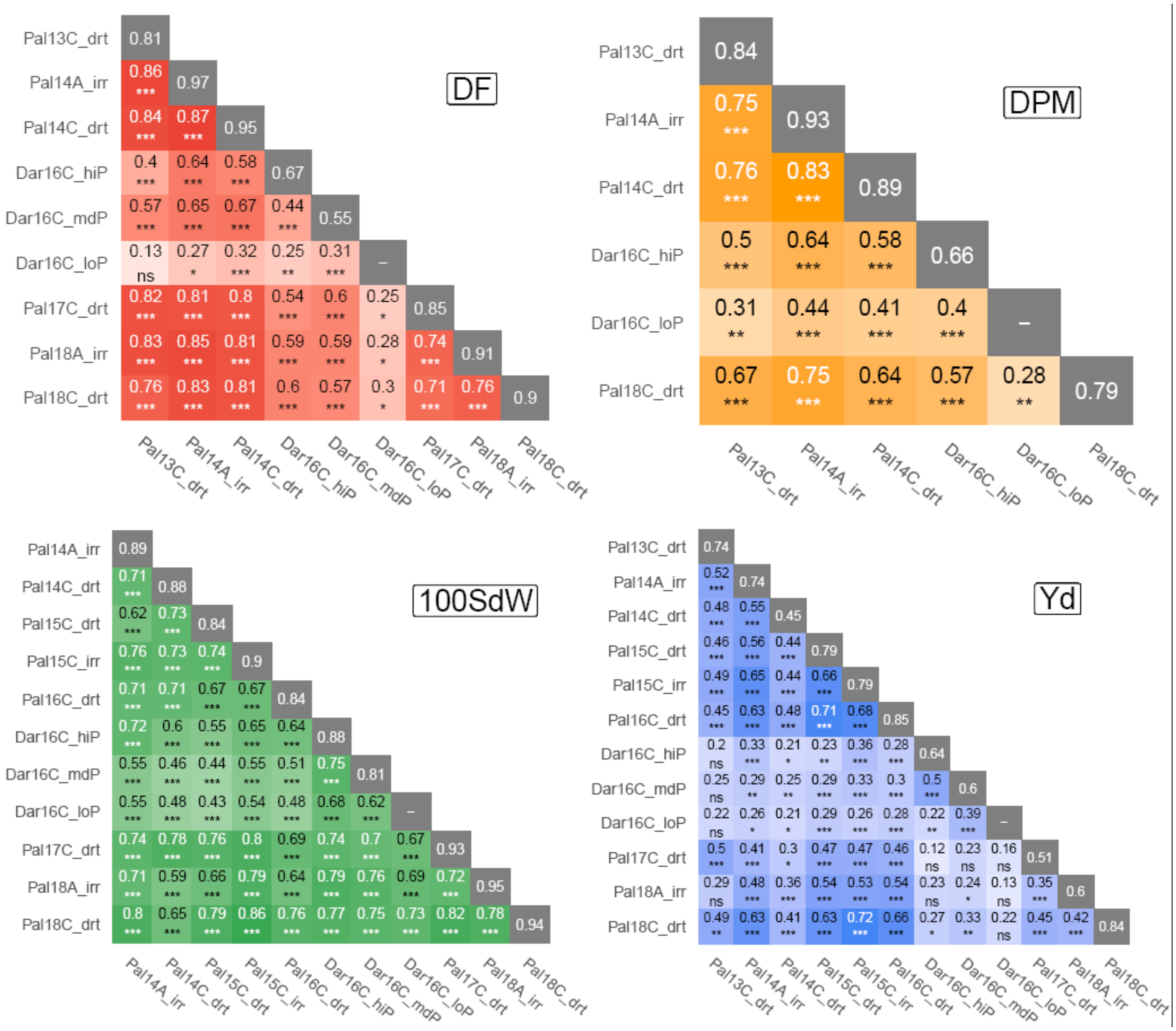

Supplementary Figure 4 Correlations of traits between trials in the VEF population evaluated between 2013 and 2018 under drought, irrigated and contrasting fertilization conditions. Days to flowering (DF), days to physiological maturity (DPM), 100 seed weight (100SdW) and yield (Yd) were evaluated in total 481 genotypes. Significance of correlations indicated as ***: $p < .0001$; **: $p < .001$; *: $p < .01$; ns = not significant. The significance p values of the correlations were corrected using the Benjamini & Hochberg adjustment for multiple tests.

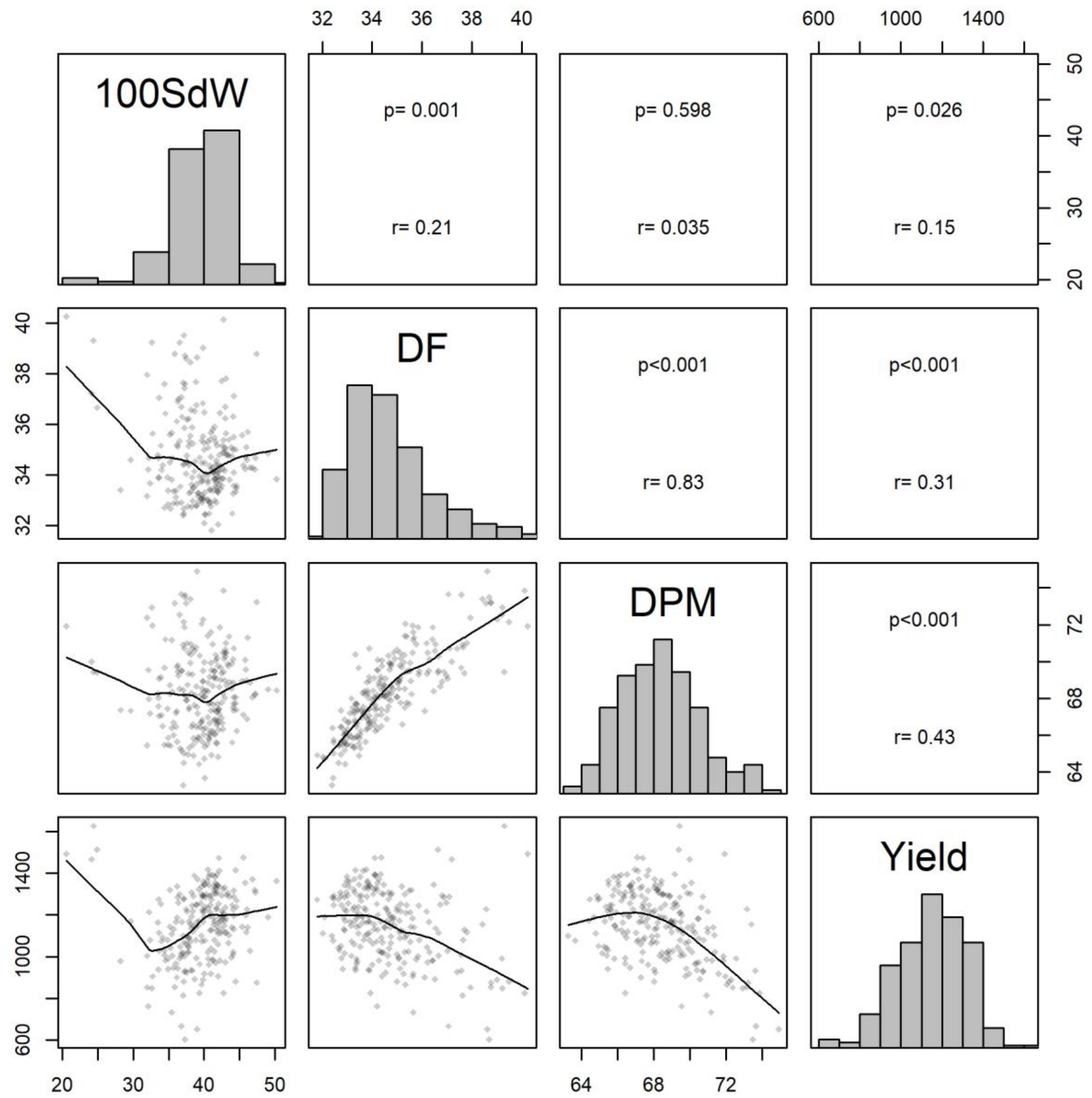

Supplementary Figure 5 Phenotypic correlations among the traits 100 seed weight (100SdW), days to flowering (DF), days to physiological maturity (DPM) and seed yield phenotypic values were adjusted over twelve trials. Black line shows the locally-weighted polynomial regression calculated by the 'lowess' function of the stats R package.

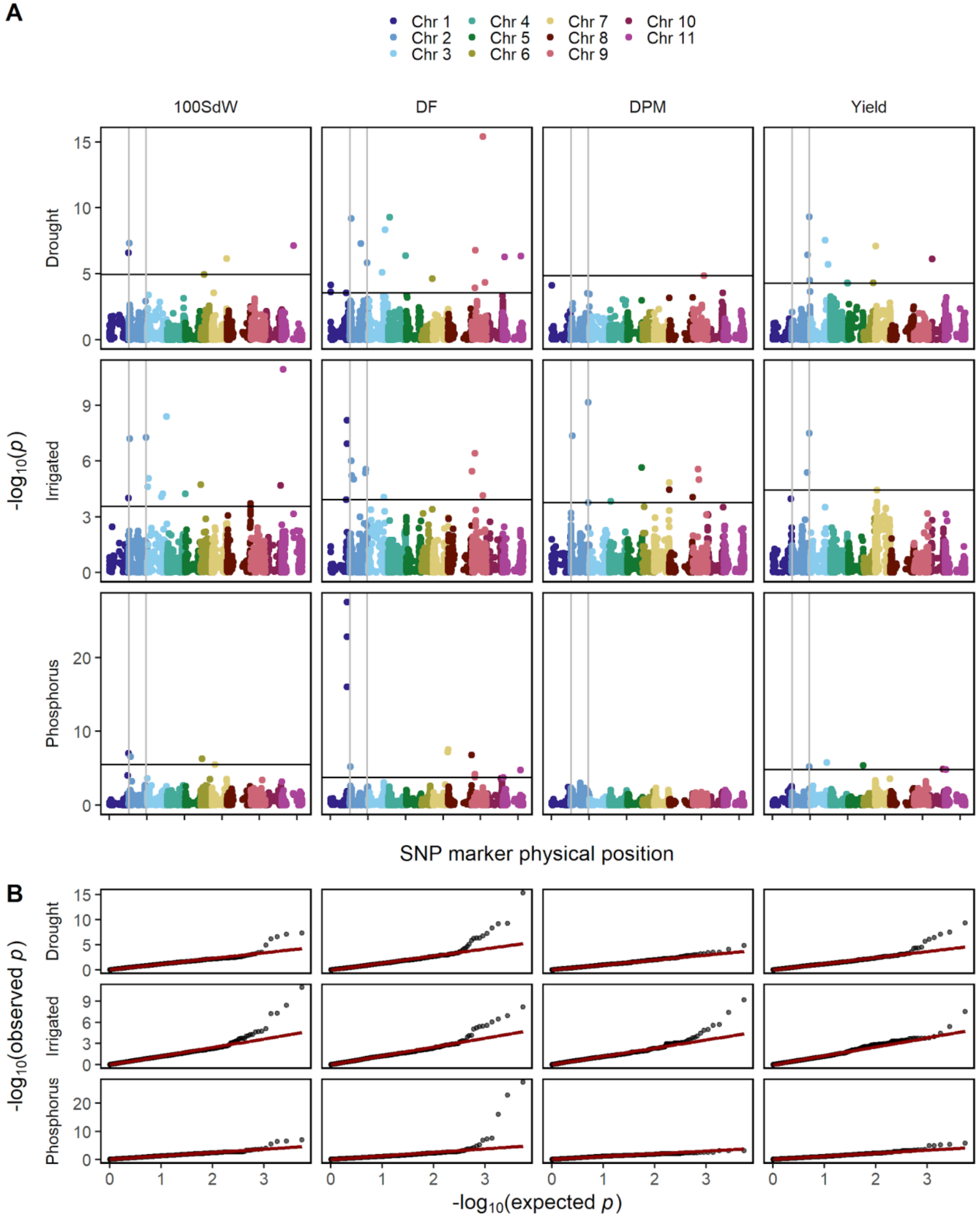

Supplementary Figure 6 Genome-wide association study for the traits 100 seed weight (100SdW), days to flowering (DF), days to physiological maturity (DPM), and seed yield in the VEF panel using best linear unbiased estimators across irrigated, drought and different phosphorus conditions. Vertical lines indicate QTL, which were found repeatedly under the different environmental conditions.

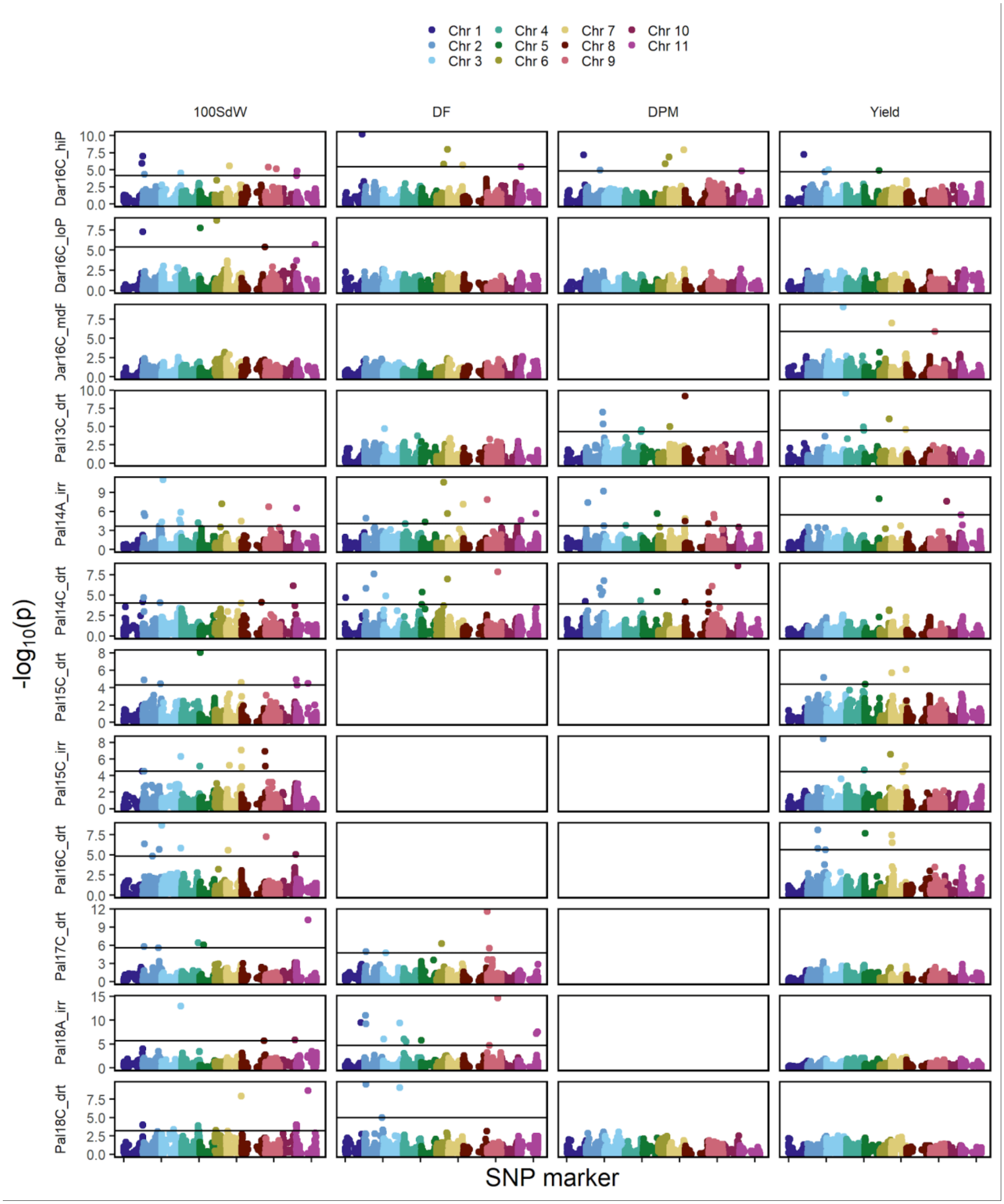

Supplementary Figure 7 Genome-wide association study for the VEF panel for each trial and for the traits 100 seed weight (100SdW), days to flowering (DF), days to physiological maturity (DPM), and seed yield.

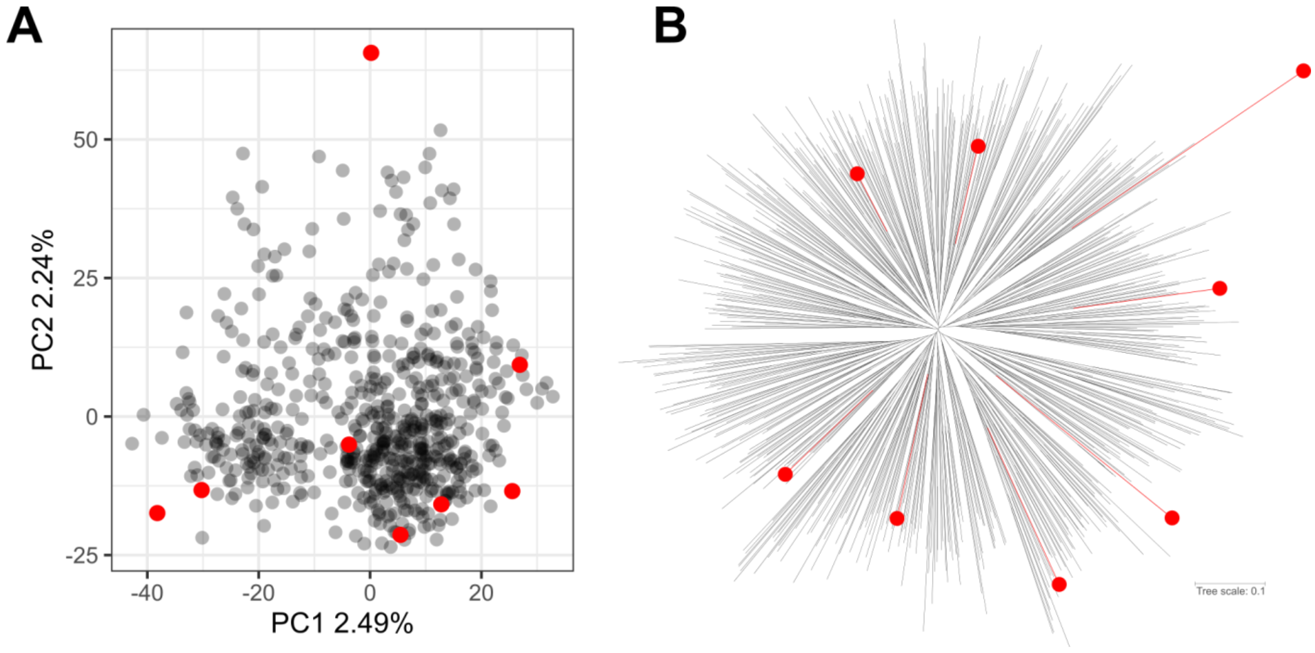

Supplementary Figure 8 Assessment of population structure for 629 MAGIC lines and 8 founders using GBS data (20,615 markers) using data from Diaz et al. (2020). (A) Location of each genotype represented by a point in the two-dimensional space defined by the eigenvectors of the first and second principal components. (B) Unrooted neighbour-joining tree. The length of the lines in the tree show the simple matching distance. The founder lines are represented by red points.

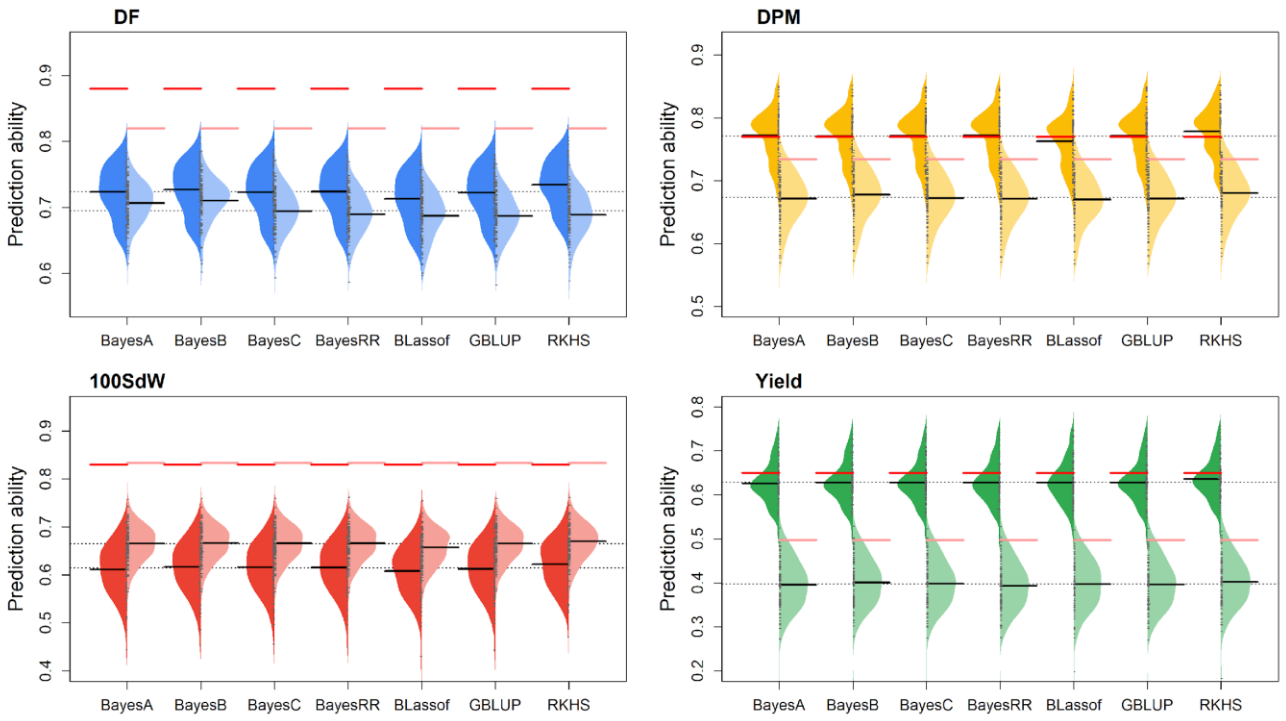

Supplementary Figure 9 Genomic prediction abilities for the traits days to flowering (DF), days to physiological maturity (DPM), 100 seed weight (100SdW) and seed yield using different parametric models on the VEF (darker colors) and on the MAGIC (lighter colors) populations. The black dotted lines show the overall prediction means for each population. The upper red line represents the broad-sense heritability for each trait and each population.

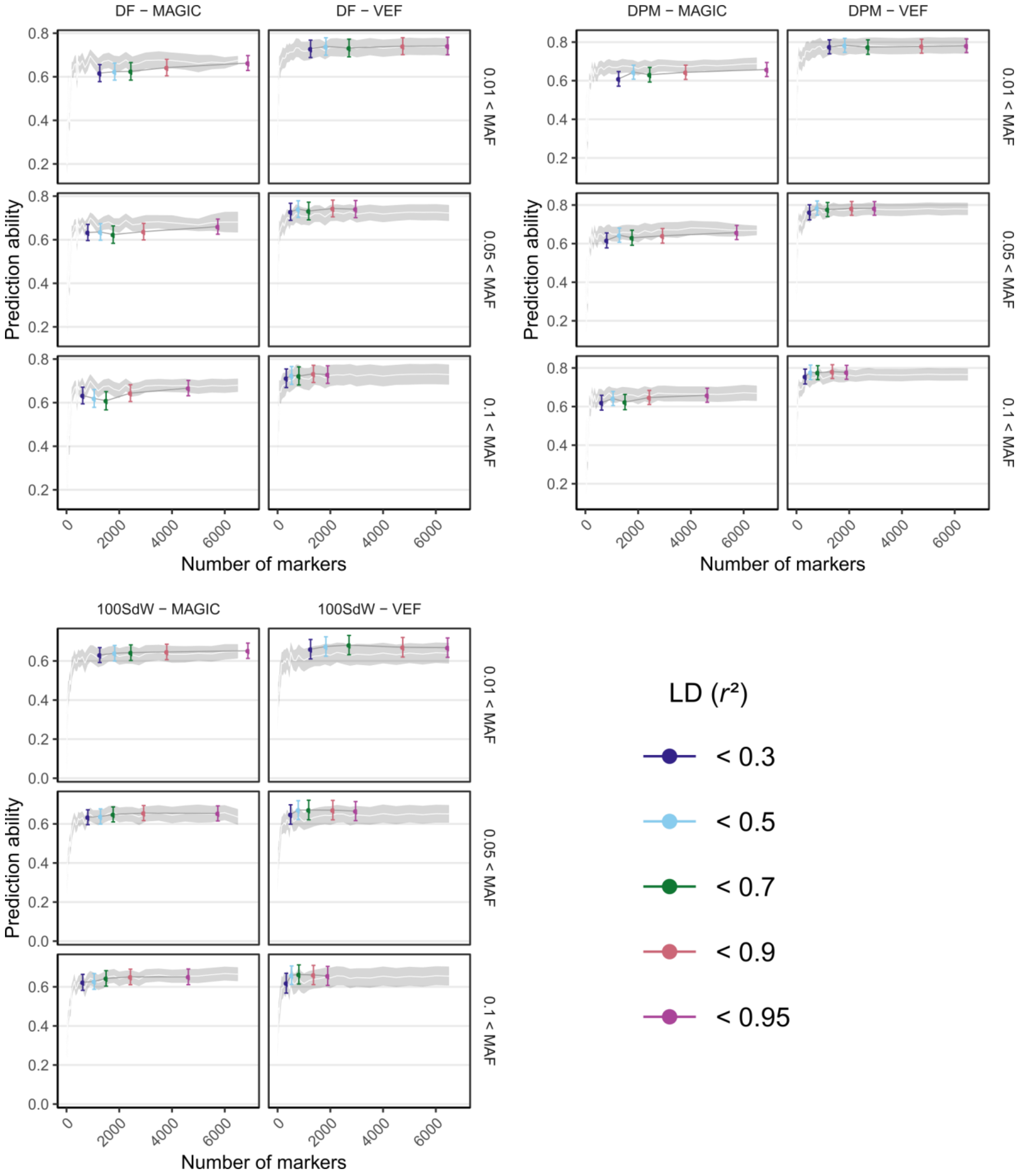

Supplementary Figure 10 Genomic prediction abilities of days to flowering (DF), days to physiological maturity (DPM), and 100 seed weight (100SdW) in response to a reduction of utilized markers. These tests were performed on the VEF and the MAGIC populations. The markers used for prediction were chosen randomly (Gray stripe, the white line shows the average prediction ability) or

based on LD and MAF parameters (colored ranges, the middle point represents the average prediction ability).

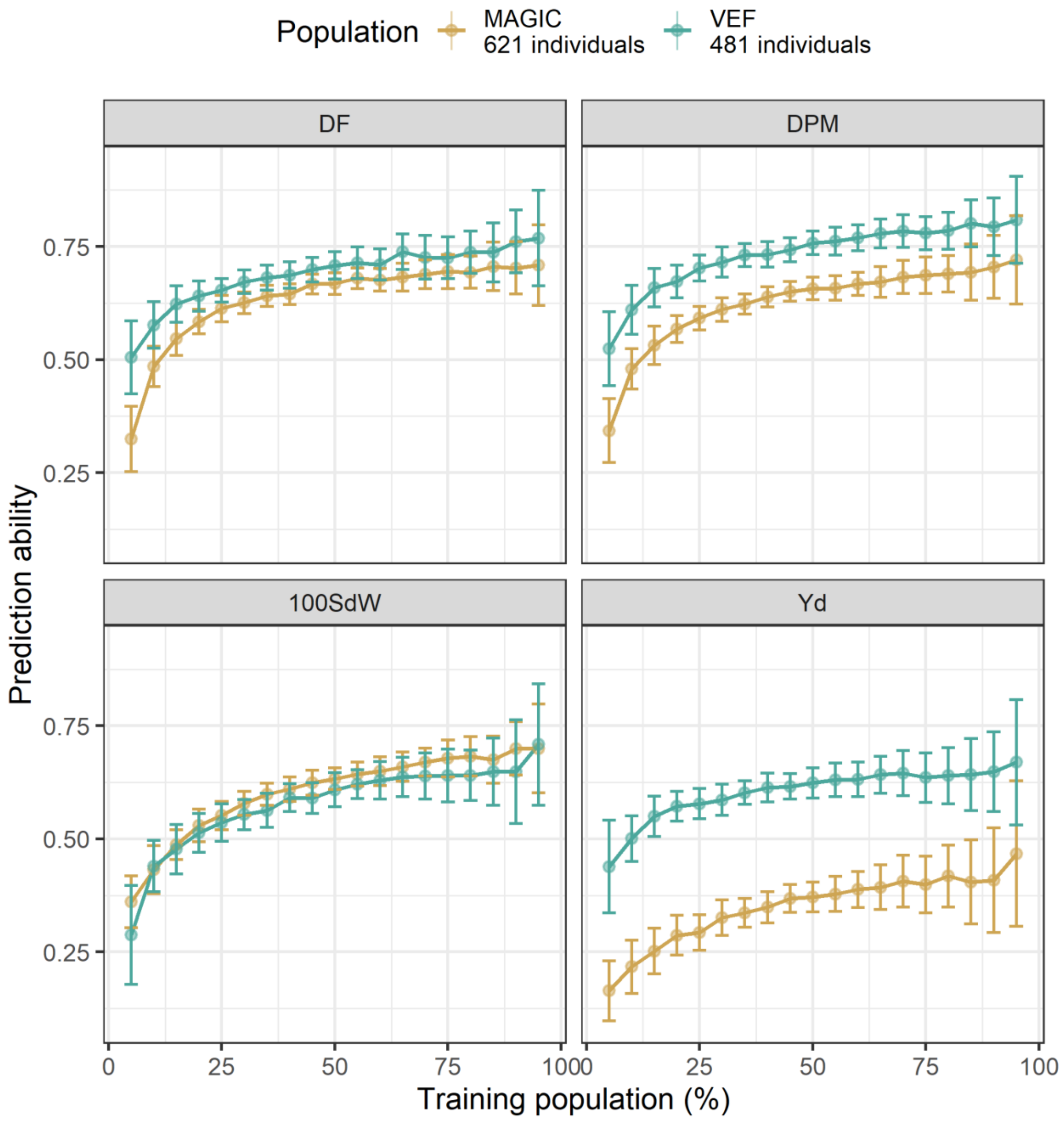

Supplementary Figure 11 Genomic prediction abilities with varying the partitioning between training and validation sets in the VEF and the MAGIC population. Days to flowering (DF), days to physiological maturity (DPM), 100 seed weight (100SdW) and yield (Yd) were evaluated.

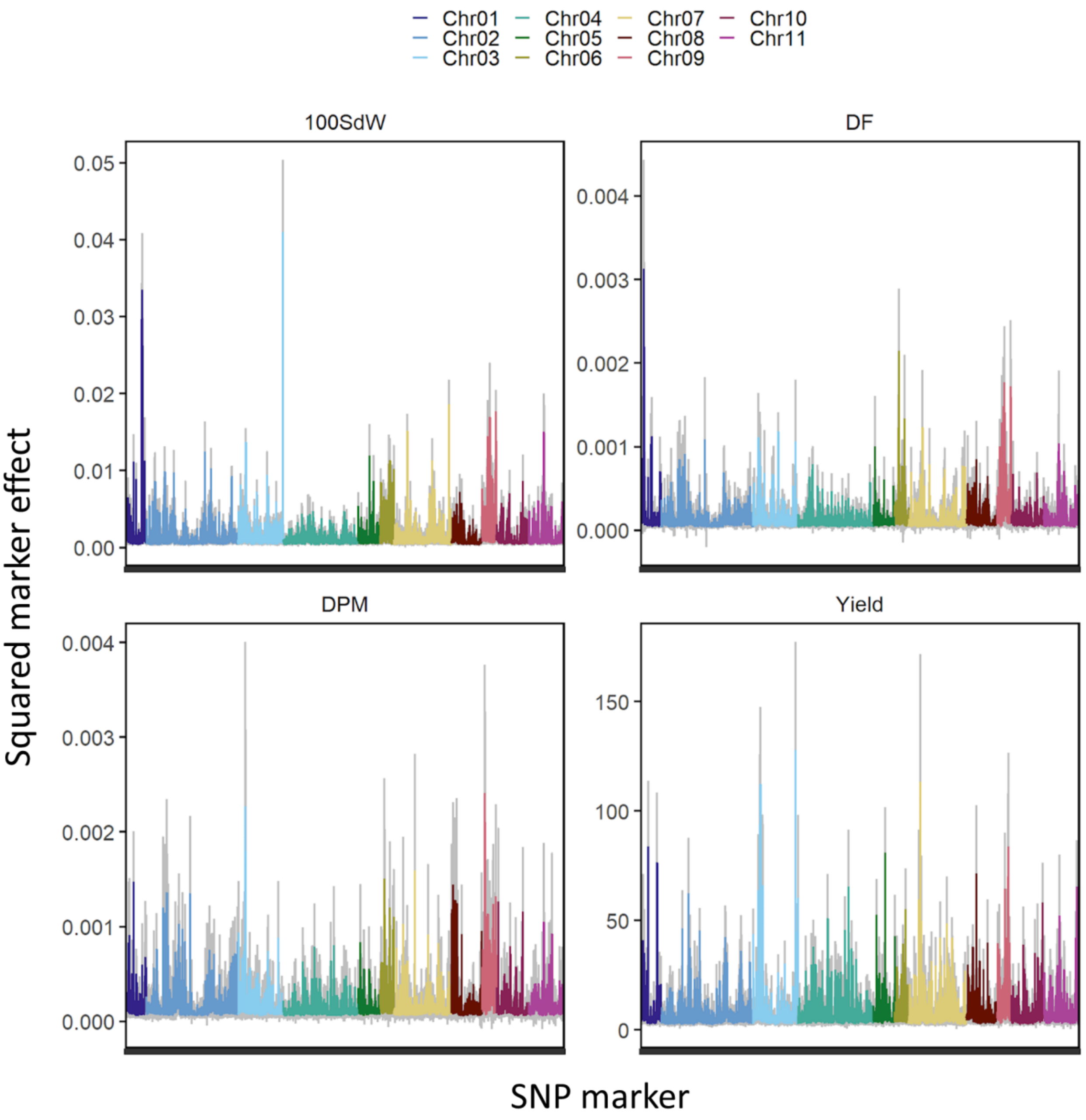

Supplementary Figure 12 The average of the squaredmarker effects for days to flowering (DF), days to physiological maturity (DPM), 100 seed weight (100SdW) and seed yield modelled over 100-fold cross validation and twelve different trials. Effects of 4962 SNP markers were estimated using Bayesian ridge regression. Grey error bars show standard deviation of the averaged effects from each trial.

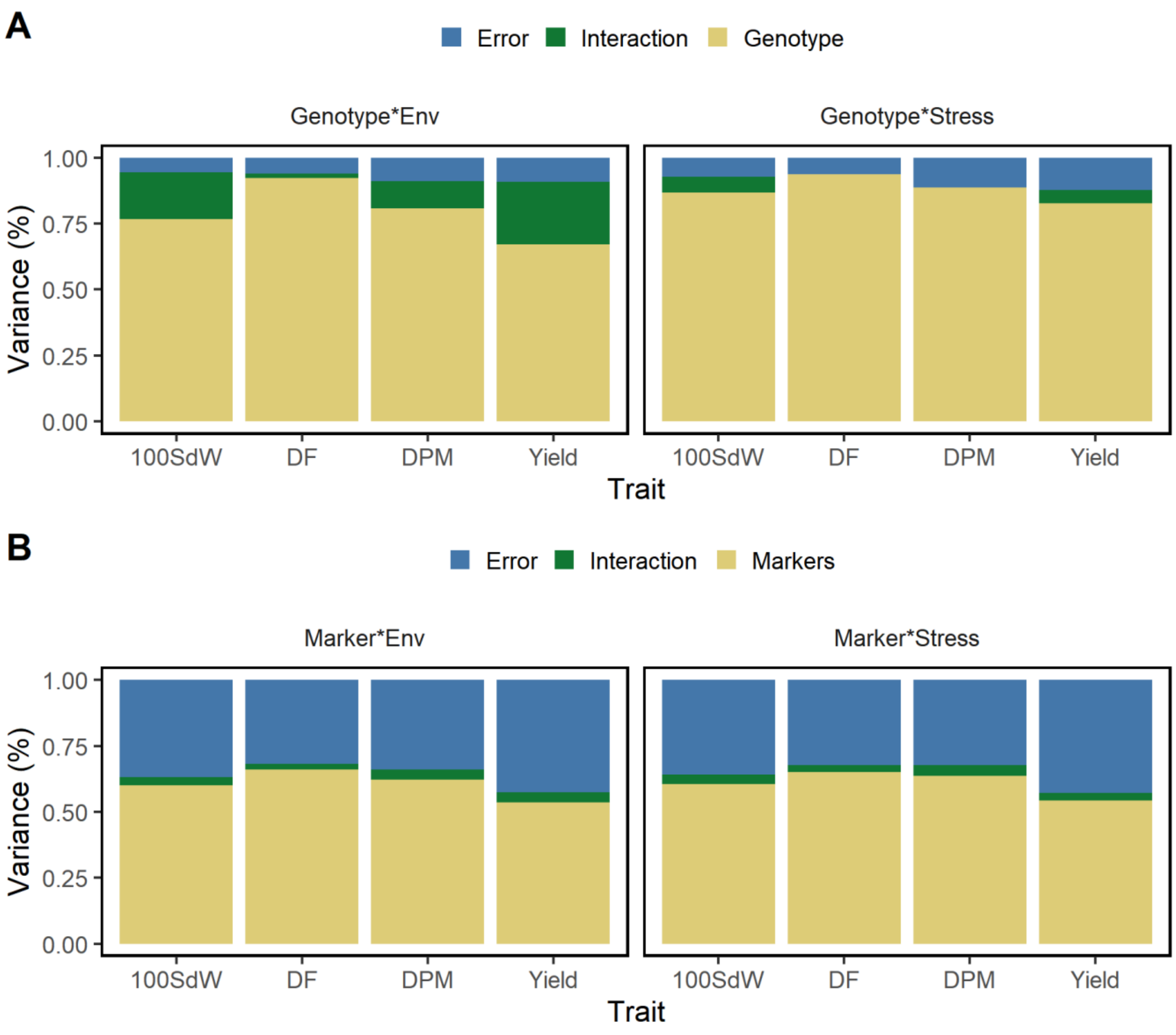

Supplementary Figure 13 Variance components of genomic models for traits evaluated in the VEF population with genotype x environment interaction. 100 seed weight (100SdW), days to flowering (DF), days to physiological maturity (DPM), and seed yield were evaluated in twelve trials between 2013 and 2018 under drought, irrigated and phosphorus fertilized conditions. In total 481 genotypes were evaluated in Darien and Palmira, Colombia. A: Variances were estimated from first-stage BLUEs. Both models were calculated as described in equation (1), however, without weights and with genotype as random factor. The Genotype*Stress used the environment term divided into stressed and non-stressed as described in equation (3). B: Variance components were derived from the genomic-estimated breeding values (GEBVs) based on the genomic heritability calculation and equation (3).

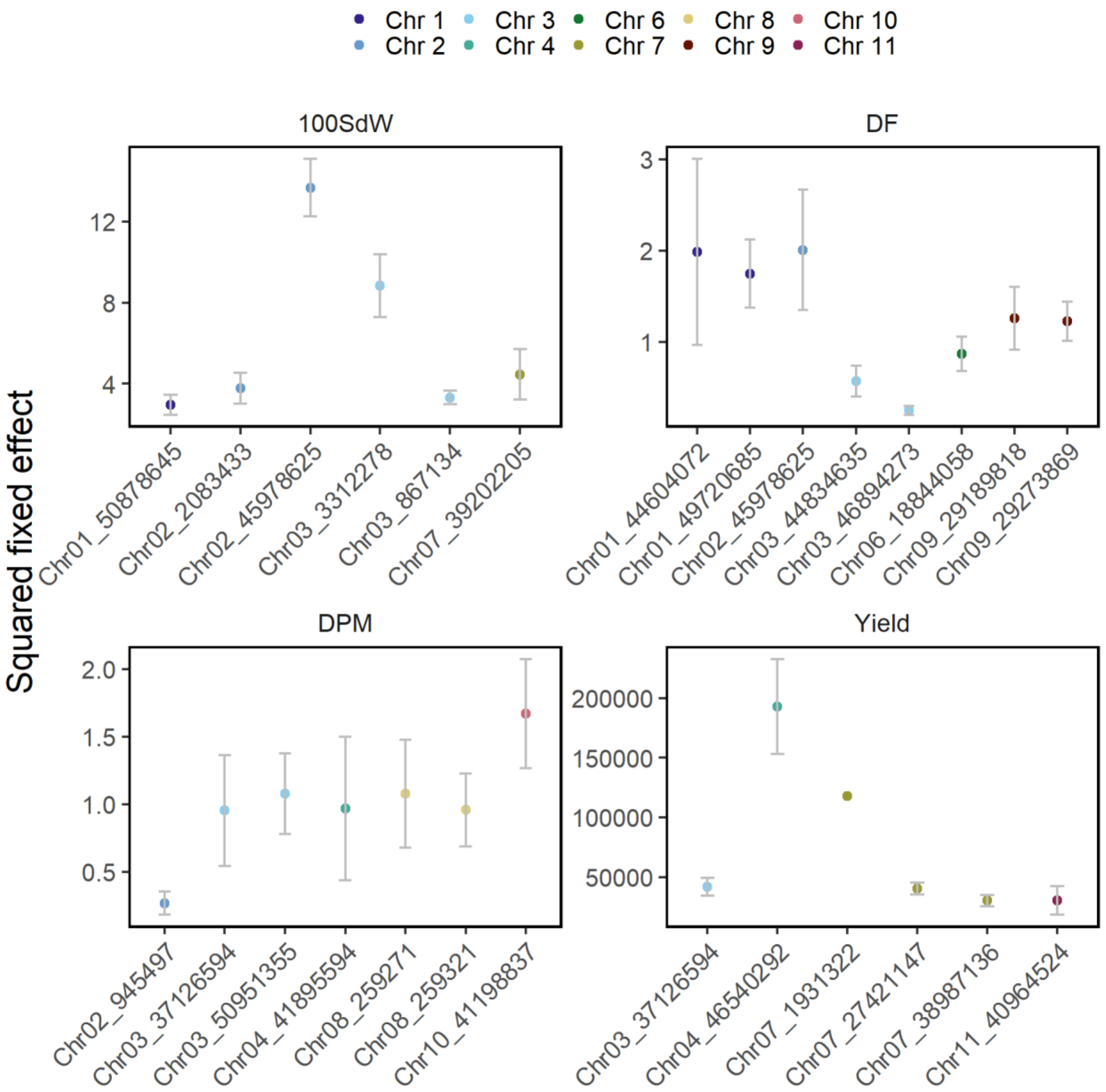

Supplementary Figure 14 The effect of markers which were identified as QTL and were included as fixed effects in the genomic prediction model (QTL model). QTL for the traits 100 seed weight (100SdW), days to flowering (DF), days to physiological maturity (DPM), and seed yield were determined in each training set and cross validation separately. QTL were detected by genome-wide association study (GWAS) on the training set using 100-fold cross-validation on second-stage best linear unbiased estimators (BLUEs)across all trials. In total, for every season 100 GWAS runs were carried out, once in each cross-validation step. Only markers which were selected more than 20 times per environment (on average) are shown. Grey error bars show the standard deviation.

2 Supplementary tables

Supplementary Table 1 Overview over all field trials planted in Palmira and Darien, Colombia.

ID	Location	Year	Tri-mester	Manag-ement	Planting date	Number of lines	Number of replicates	Plot area (m^2)
Pal13C_drt	Palmira	2013	C	Drought	7/4/2013	156	3	2.22
Pal14A_irr	Palmira	2014	A	Irrigated	1/28/2014	265	3	4.44
Pal14C_drt	Palmira	2014	C	Drought	7/26/2014	299	2	4.44
Pal15C_drt	Palmira	2015	C	Drought	7/29/2015	344	3	2.22
Pal15C_irr	Palmira	2015	C	Irrigated	7/29/2015	345	3	2.22
Pal16C_drt	Palmira	2016	C	Drought	8/4/2016	345	3	2.22
Dar16C_hiP	Darien	2016	C	Hi P	5/4/2016	345	2	1.98
Dar16C_loP	Darien	2016	C	Low P	5/4/2016	346	1	1.98
Dar16C_mdP	Darien	2016	C	Medium P	5/4/2016	346	2	1.98
Pal17C_drt	Palmira	2017	C	Drought	7/20/2017	264	3	4.44
Pal18A_irr	Palmira	2018	A	Irrigated	2/12/2018	265	3	2.22
Pal18C_drt	Palmira	2018	C	Drought	8/17/2018	175	3	2.22

Supplementary Table 2 Different phosphorus (P) and organic matter content in the three field trials in Darien

	Low P	Medium P	High P
pH	5.18	4.99	5.42
P content (mg/kg)	9.94	16.03	26.53
Organic material (%)	5.68	5.3	6.43

Supplementary Table 3 Significant QTL effects identified by genome-wide association study in best linear unbiased estimators (BLUEs) over all seasons using BLINK algorithm implemented in GAPIT.

Variable	Marker	Chr	Position	P value
100SdW	Chr02_4652129	2	4652129	1.02E-06
100SdW	Chr03_19501	3	19501	1.73E-05
100SdW	Chr05_967160	5	967160	1.32E-05
100SdW	Chr07_7517898	7	7517898	2.05E-05
100SdW	Chr11_2160618	11	2160618	2.69E-05
DF	Chr01_49720685	1	49720685	6.54E-08
DF	Chr02_27129356	2	27129356	5.80E-07
DF	Chr02_45978625	2	45978625	1.20E-05
DF	Chr04_3399867	4	3399867	4.00E-07
DF	Chr06_18844058	6	18844058	1.75E-06
DF	Chr07_3845608	7	3845608	1.88E-05
DF	Chr07_29487776	7	29487776	4.99E-05
DF	Chr08_62188128	8	62188128	0.000152883
DF	Chr09_29268261	9	29268261	1.08E-06
DF	Chr11_49117257	11	49117257	5.54E-07
DPM	Chr02_46017330	2	46017330	6.05E-09
DPM	Chr04_46203242	4	46203242	2.90E-07
Yield	Chr02_46424036	2	46424036	3.06E-06
Yield	Chr07_37961682	7	37961682	6.03E-06
Yield	Chr07_38824916	7	38824916	8.52E-06
Yield	Chr07_39433002	7	39433002	6.89E-05
